# Supplementary material for: Identifying proteomic risk factors for cancer using prospective and exome analyses of 1463 circulating proteins and risk of 19 cancers in the UK Biobank
Source: Nat Commun. 2024 May 15;15:4010. doi: 10.1038/s41467-024-48017-6 (PMC11096312; doi:10.1038/s41467-024-48017-6)
Supplement: Supplementary file 1 — Supplementary Information [file 41467_2024_48017_MOESM1_ESM.pdf]

Contents

Supplementary Methods: cancer specific adjustments 3
Supplementary Methods: Pathway analyses 5
Supplementary Figure 1. Study design flow chart and results summary 6
Supplementary Figure 2. Percentage change in the log hazard ratios between fully and minimally adjusted models 7
Supplementary Figure 3. Pathway analysis for ENT significant protein-cancer associations across Gene-Ontology Biological Processes 8
Supplementary Figure 4. Pathway analysis for ENT significant protein-cancer associations across Gene-Ontology Cellular Component 9
Supplementary Figure 5. Pathway analysis for ENT significant protein-cancer associations across Gene-Ontology Molecular Function 10
Supplementary Figure 6. Descriptive summary of expression for protein identified to associate with cancer risk by cell and tissue type 11
Supplementary Figure 7. Volcano plot for the prospective association of circulating proteins with risk of head and neck cancer 12
Supplementary Figure 8. Volcano plot for the prospective association of circulating proteins with risk of oral cancer 13
Supplementary Figure 9. Volcano plot for the prospective association of circulating proteins with risk of oesophagus cancer 14
Supplementary Figure 10. Volcano plot for the prospective association of circulating proteins with risk of oesophageal adenocarcinoma 15
Supplementary Figure 11. Volcano plot for the prospective association of circulating proteins with risk of stomach cancer 16
Supplementary Figure 12. Volcano plot for the prospective association of circulating proteins with risk of colorectum cancer 17
Supplementary Figure 13. Volcano plot for the prospective association of circulating proteins with risk of colon cancer 18
Supplementary Figure 14. Volcano plot for the prospective association of circulating proteins with risk of rectal cancer 19
Supplementary Figure 15. Volcano plot for the prospective association of circulating proteins with risk of liver cancer 20
Supplementary Figure 16. Volcano plot for the prospective association of circulating proteins with risk of lung cancer 21
Supplementary Figure 17. Volcano plot for the prospective association of circulating proteins with risk of lung adenocarcinoma 22
Supplementary Figure 18. Volcano plot for the prospective association of circulating proteins with risk of lung squamous cell carcinoma 23

Supplementary Figure 19. Volcano plot for the prospective association of circulating proteins with risk of lung small cell carcinoma 24
Supplementary Figure 20. Volcano plot for the prospective association of circulating proteins with risk of breast cancer 25
Supplementary Figure 21. Volcano plot for the prospective association of circulating proteins with risk of prostate cancer 26
Supplementary Figure 22. Volcano plot for the prospective association of circulating proteins with risk of kidney cancer 27
Supplementary Figure 23. Volcano plot for the prospective association of circulating proteins with risk of bladder cancer 28
Supplementary Figure 24. Volcano plot for the prospective association of circulating proteins with risk of brain cancer 29
Supplementary Figure 25. Volcano plot for the prospective association of circulating proteins with risk of non-Hodgkin lymphoma 30
Supplementary Figure 26. Volcano plot for the prospective association of circulating proteins with risk of diffuse lymphoma 31
Supplementary Figure 27. Volcano plot for the prospective association of circulating proteins with risk of multiple myeloma 32
Supplementary Figure 28. Volcano plot for the prospective association of circulating proteins with risk of leukemia 33
Supplementary Figure 29. Volcano plot for the prospective association of circulating proteins with risk of ovarian cancer 34
Supplementary Figure 30. Volcano plot for the prospective association of circulating proteins with risk of endometrial cancer 35
Supplementary Figure 31. Volcano plot for the prospective association of circulating proteins with risk of thyroid cancer 36

Supplementary Methods: cancer specific adjustments

All cancer-specific multivariable regression models were stratified by age and sex and adjusted for region and Townsend deprivation index (minimally adjusted model) and additionally adjusted for the following for:

Bladder cancer - [smoking status and number of cigarettes smoked (8 categories: never, former smoker and < 15 cigarettes/day, former smoker and ≥15 cigarettes/day, former smoker and number of cigarettes smoked unknown, current smoker and < 15 cigarettes/day, current smoker and ≥15 cigarettes/day, current smoker and number of cigarettes smoked unknown, unknown), cigarette-pack years (5 categories: quintiles, unknown), and BMI (4 categories <25, 25-29, 30-34, ≥35 kg/m^2)]; for

Breast cancer - [family history of breast cancer (yes, no or unknown), parity and age at first birth (11 categories: ≥3 kids, <25 years, ≥3 kids, 25-29 years, ≥3 kids, ≥30 years, ≥3 kids, age not reported, : 1-2 kids, <25 years, 1-2 kids, 25-29 years, 1-2 kids, ≥30 years, 1-2 kids, age not reported, no children, not applicable, men), age menarche (6 categories: <11 years, 12-13 years, 14-15 years, ≥16 years, men, unknown), hormone replacement therapy use (4 categories: never, past, men, unknown), oral contraceptive use (5 categories: never, for <20 years, for ≥20 years, unknown, men), alcohol intake (6 categories: < 1 g/day, 1-9 g/day, 10-19 g/day, ≥20 g/day, non-drinkers, unknown), physical activity (6 categories: <10 metabolic equivalent (MET) hours per week, 10-19 MET hours per week, 20-39 MET hours per week, 40-59 MET hours per week, ≥60 MET hours per week, unknown), BMI (4 categories), menopausal status (4 categories: pre-menopausal, post-menopausal, men, unknown), smoking status (6 categories: never, former smoker, current smoker, <15 cigarettes/day, current smoker, ≥15 cigarettes/day, current smoker, number of cigarettes unknown, unknown), and an interaction between BMI (4 categories) and menopausal status (4 categories)]; for

Head and neck cancer (including subtypes) and liver cancer - [smoking status (6 categories: never, former smoker, current smoker, <15 cigarettes/day, current smoker, ≥15 cigarettes/day, current smoker, number of cigarettes unknown, unknown), BMI (4 categories), and alcohol intake (6 categories)]; for

Kidney cancer - [smoking status and number of cigarettes smoked (8 categories), cigarette-pack years (5 categories), and BMI (4 categories)]; for

Leukemia - [smoking status and number of cigarettes smoked (8 categories)]; for

Lung cancer (including subtypes) - [family history of lung cancer (yes, no or unknown), smoking status and number of cigarettes smoked (8 categories), smoking status and years of smoking (9 categories: never, former smoker and < 30 years, former smoker and ≥30 years, former smoker and number of cigarettes smoked unknown, current smoker and < 30 years, current smoker and ≥ 30 years, current smoker and number of cigarettes smoked unknown, unknown status, unknown), and particulate matter (5 categories: quintiles, unknown)]; for

Melanoma - [use sun or UV protection (6 categories: never/rarely, sometimes, most of the time, always, do not go out in the sunshine, unknown), hours spent outdoors on a summer day (continuous), ease of skin tanning (5 categories: never tan only burn, get mildly or occasionally tanned, get moderately tanned, get very tanned, unknown)]; for

Colorectal cancer and its subtypes - [family history of colorectal cancer (yes, no or unknown), waist to hip ratio (6 categories: quintiles, unknown), frequency of processed meat intake (< once per week, ≥ once per week, unknown), physical activity (6 categories), BMI (4 categories), alcohol intake (6 categories), and smoking status and years of smoking (9 categories)]; for

Ovarian cancer - [family history of breast cancer (yes, no or unknown), parity and age at first birth (11 categories), age menarche (6 categories), hormone replacement therapy use (4 categories), oral contraceptive use (5 categories, smoking status and years of smoking (9 categories), BMI (4 categories), menopausal status (4 categories), and an interaction term between BMI (4 categories) and menopausal status (4 categories)]; for

Pancreas cancer - [smoking status and number of cigarettes smoked (8 categories), and BMI (4 categories)];

Prostate cancer - [family history of prostate cancer (yes, no or unknown), and body mass index (BMI) (4 categories); for

Stomach cancer - [BMI (4 categories), smoking status (6 categories: never, former smoker, current smoker, <15 cigarettes/day, current smoker, ≥15 cigarettes/day, current smoker, number of cigarettes unknown, unknown) and alcohol intake (6 categories)]; for

Thyroid cancer - [BMI (4 categories)]; and for

Uterine cancer [parity and age at first birth (11 categories), age menarche (6 categories), hormone replacement therapy use (4 categories), oral contraceptive use (5 categories, physical activity (6 categories), smoking status and years of smoking (9 categories), BMI (4 categories), and menopausal status (4 categories)].

We did not adjust brain cancer and the blood cancer sub-types other than leukemia analyses beyond the minimally adjusted model since there were no known relevant confounders in this dataset.

Supplementary Methods: Pathway analyses

We completed a pathway enrichment analysis for proteins identified as passing ENT-significance for each cancer separately using the clusterProfiler package (version 3.18.1). Specifically, we used the enricher() function to identify set of proteins that were overrepresented in annotations from Gene Ontology (GO) biological processes (BP), cellular component (CC), and molecular functions (MF). Significance was defined using the Benjamini-Hochberg method ($p < 0.05$).

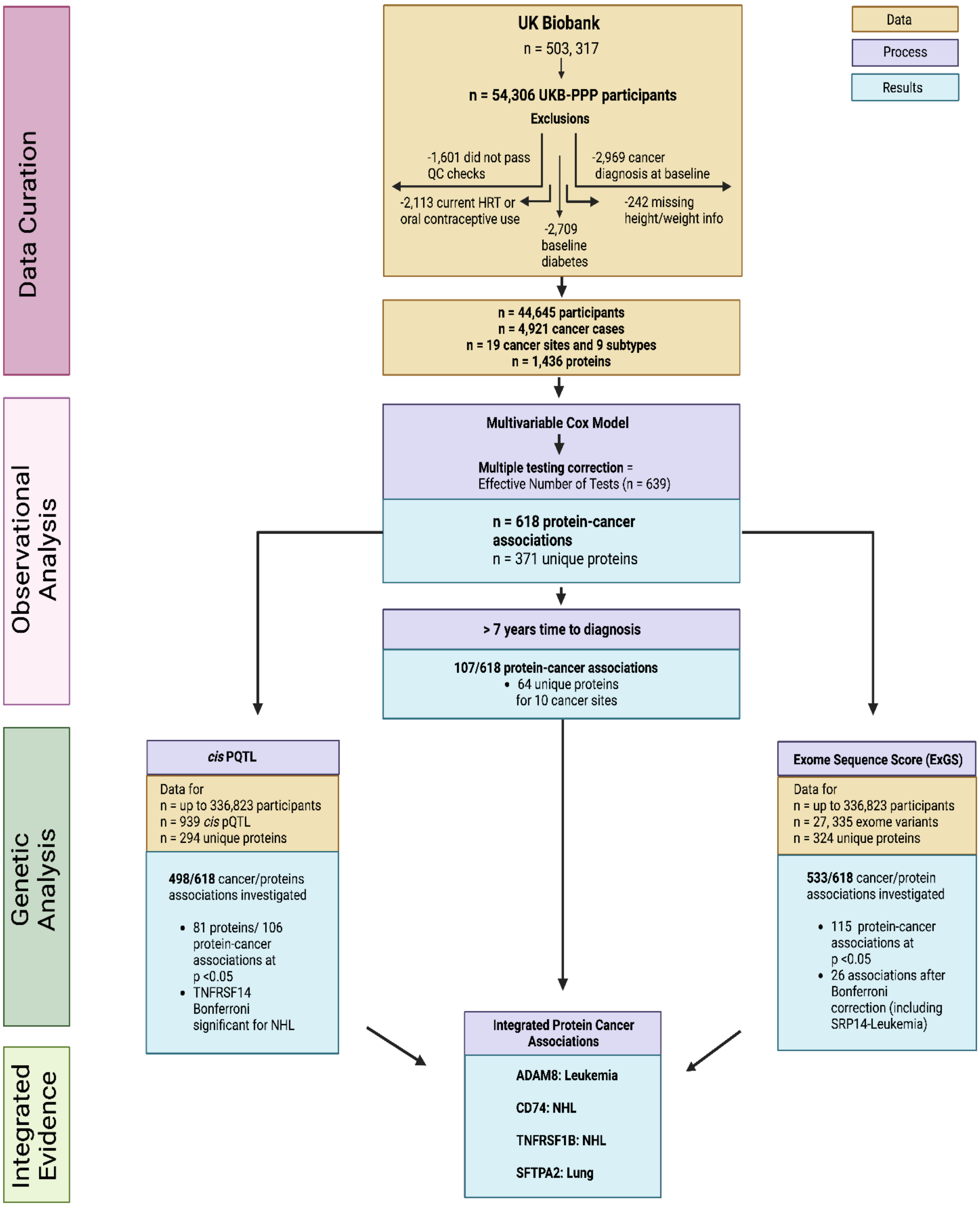

Supplementary Figure 1. Study design flow chart and results summary

Multivariable Cox Model was performed on individuals that were part of the UK Biobank Pharma Proteomics Project (UKB-PPP) after excluding individuals that failed QC, had missing data, were diabetic or were using hormone replacement therapy (HRT) or using oral conceptive use at baseline. Genetic evidence using cis-pQTLs and exome sequencing scoring (ExGS) for protein-cancer associations was investigated. Lastly, data was integrated to investigate protein-cancer associations with multiple levels of support.

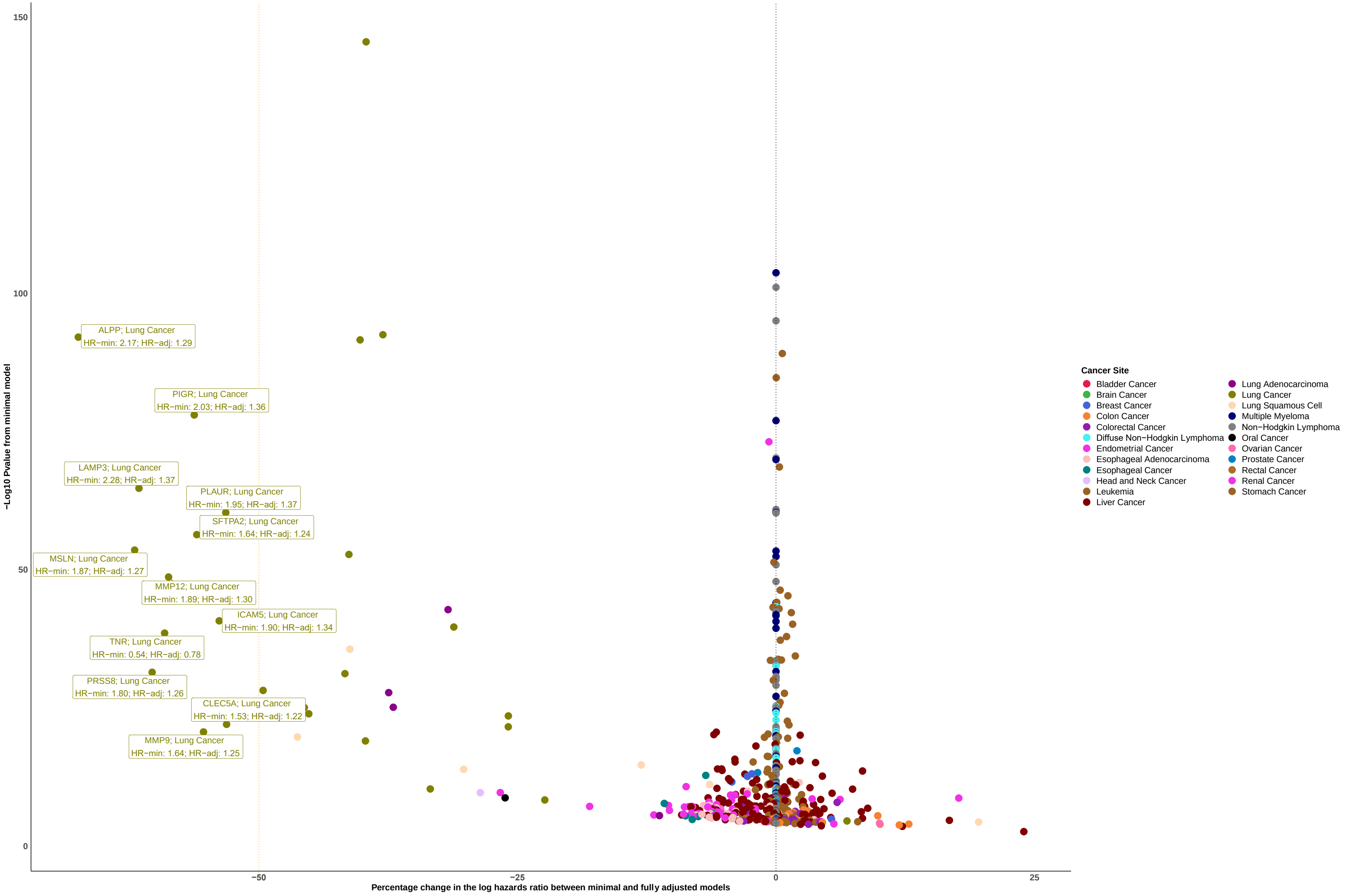

Supplementary Figure 2. Percentage change in the log hazard ratios between fully and minimally adjusted models

This figure displays the differences between the minimally model and fully adjusted models with the x-axis displaying the percentage change with the y-axis displaying the -log10(P-value) from the minimal model. The colours represent the cancer site. Source data are provided as a Source Data file.

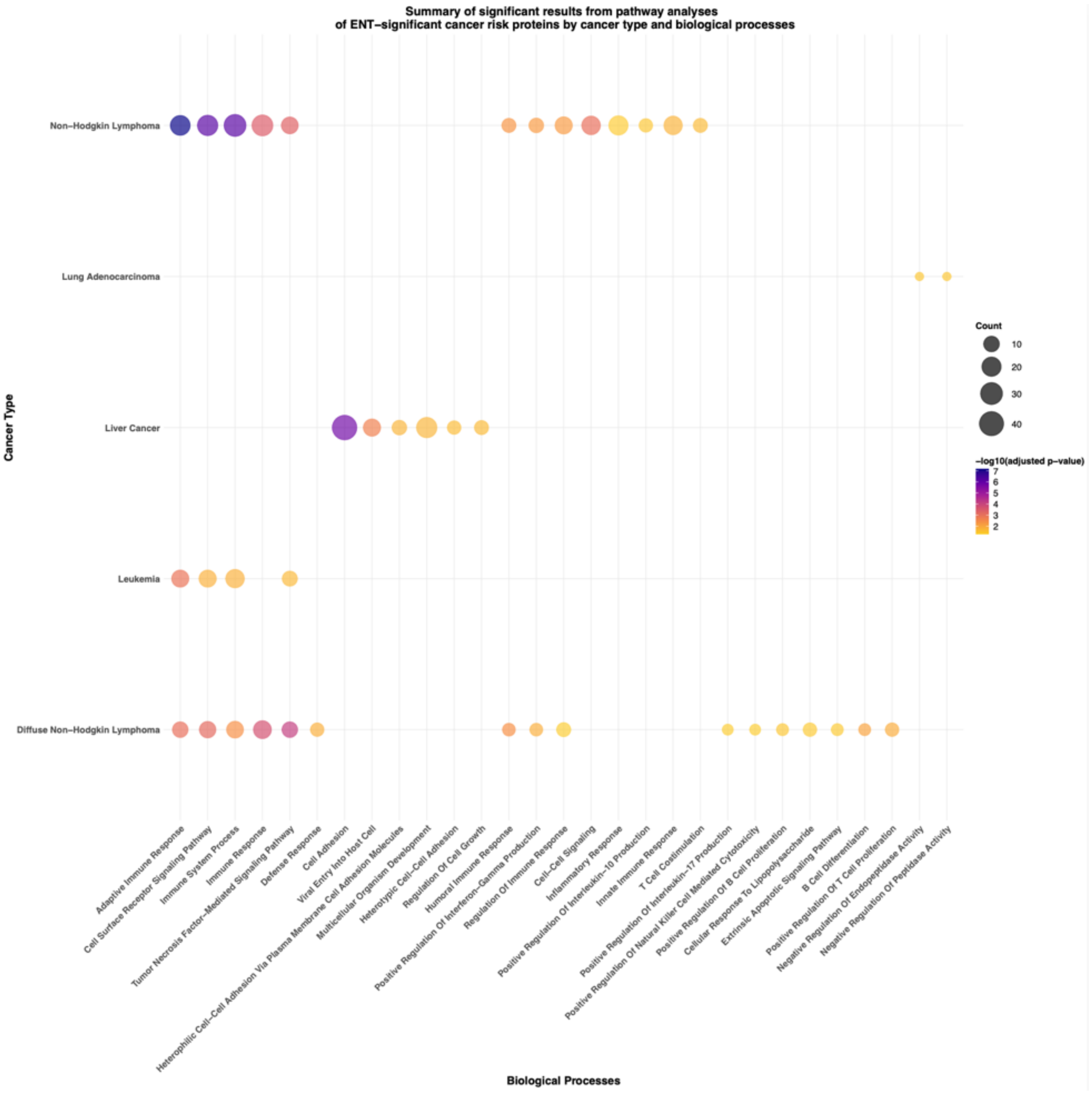

Supplementary Figure 3. Pathway analysis for ENT significant protein-cancer associations across Gene-Ontology Biological Processes

This figure displays pathways that were significantly enriched for biological processes based on ENT-significant protein-cancer associations. The x-axis displays the identified biological processes and the y-axis displays the cancer sites. The colours represent the significance of pathway results and the point size represents the count of overlapping proteins between pathway and protein-cancer associations.

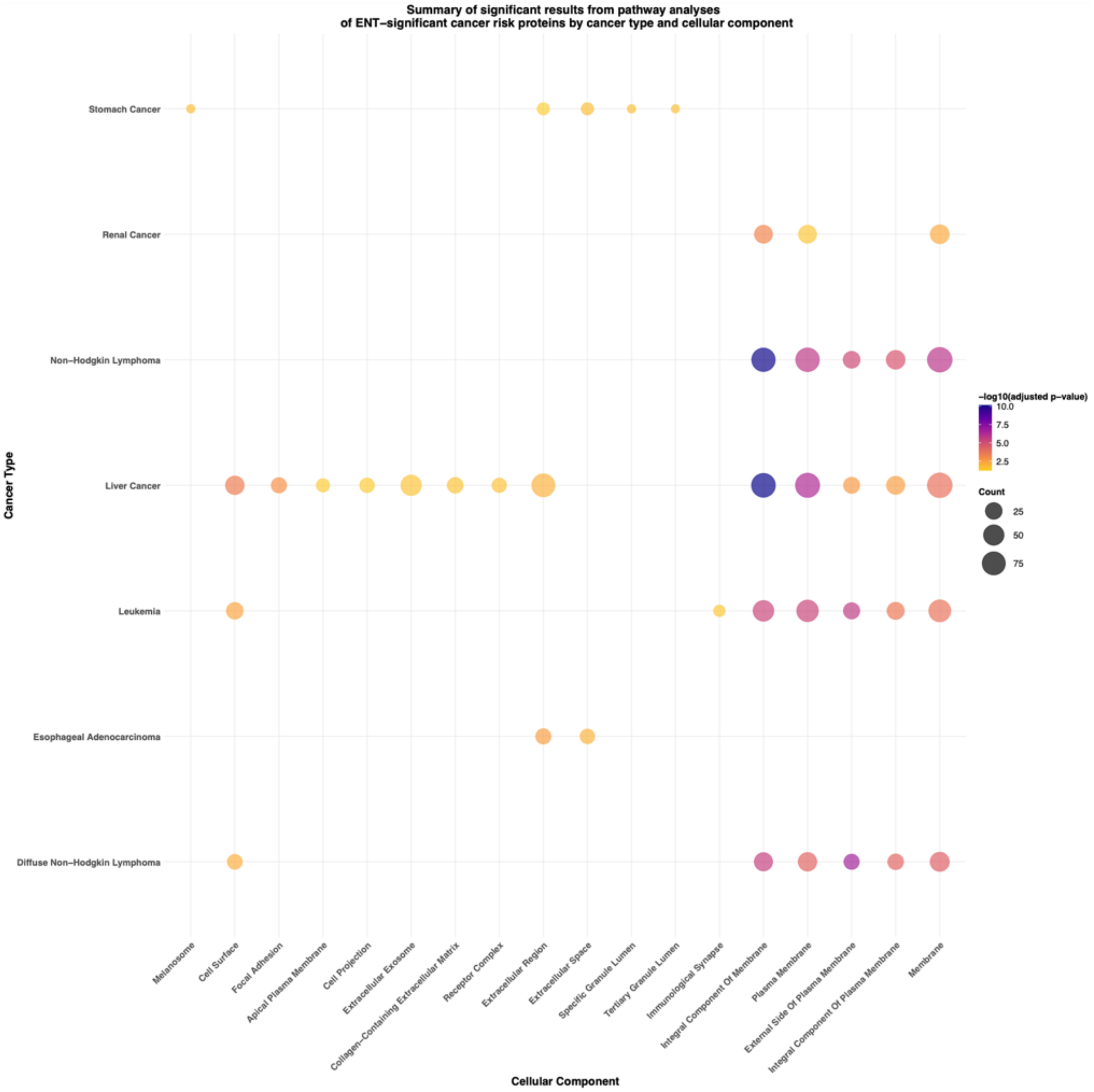

Supplementary Figure 4. Pathway analysis for ENT significant protein-cancer associations across Gene-Ontology Cellular Component

This figure displays pathways that were significantly enriched for cellular component based on ENT-significant protein-cancer associations. The x-axis displays the identified biological processes and the y-axis displays the cancer sites. The colours represent the significance of pathway results and the point size represents the count of overlapping proteins between pathway and protein-cancer associations.

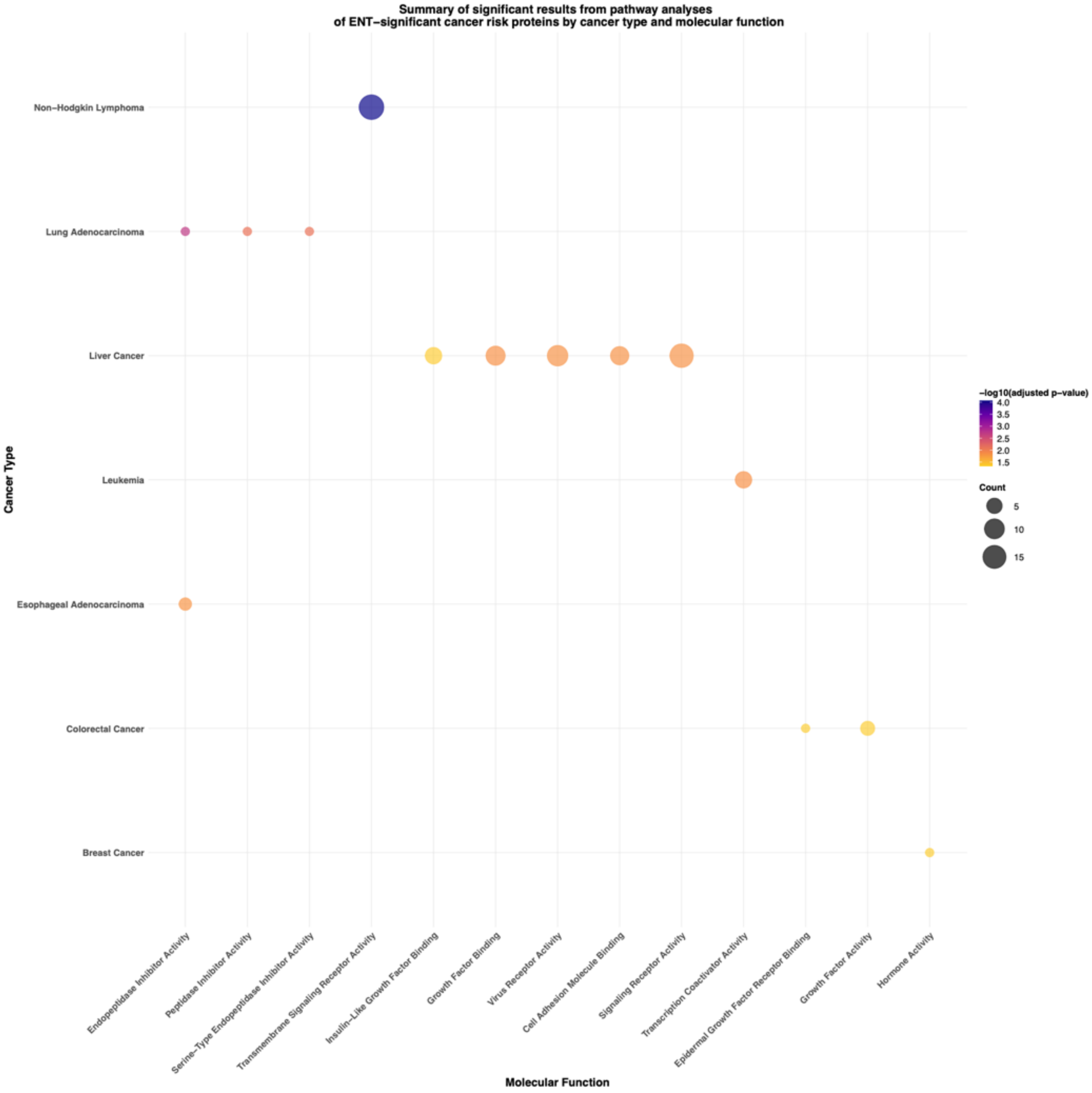

Supplementary Figure 5. Pathway analysis for ENT significant protein-cancer associations across Gene-Ontology Molecular Function

This figure displays pathways that were significantly enriched for molecular function based on ENT-significant protein-cancer associations. The x-axis displays the identified biological processes and the y-axis displays the cancer sites. The colours represent the significance of pathway results and the point size represents the count of overlapping proteins between pathway and protein-cancer associations.

A.

B.

C.

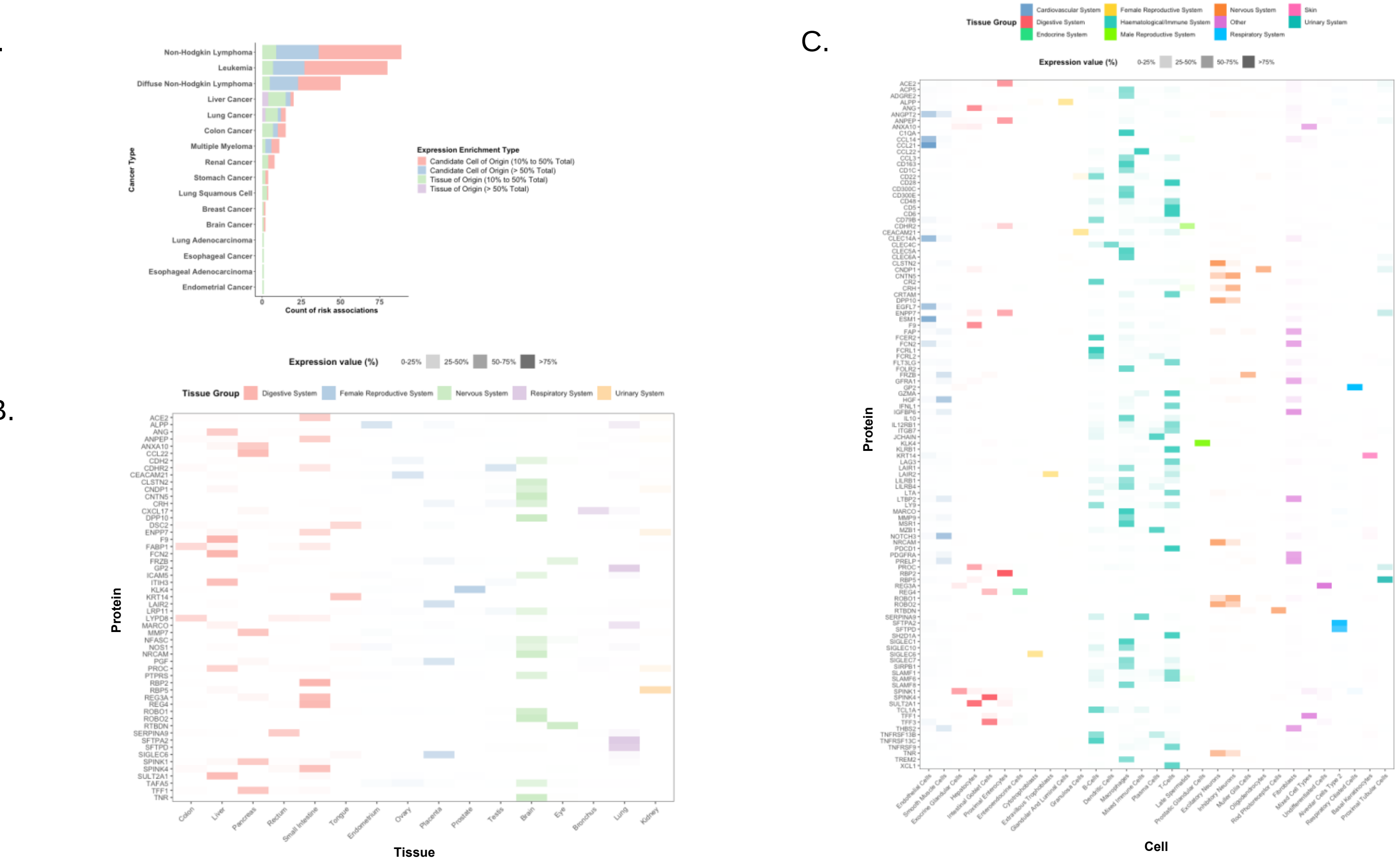

Supplementary Figure 6. Descriptive summary of expression for protein identified to associate with cancer risk by cell and tissue type

This set of figures displays the enrichment of mRNA expression at the cellular and tissue level for cancer risk proteins: a) summarizes the count of proteins that associate with cancer risk and whose genes are either enriched for expression (between 10% and 50% of total expression) or majority expressed (greater than 50% of total expression) on the candidate cell or tissue of cancer origin by cancer site; b) displays the cross-tissue mRNA expression of the genes that code for proteins associated with cancer risk that are also majority expressed in one tissue; c) displays the cross-cellular mRNA expression of the genes that code for proteins associated with cancer risk that are also majority expressed in one cell. Both b) tissues and b) cells are grouped by higher-order organ systems. Source data are provided as a Source Data file.

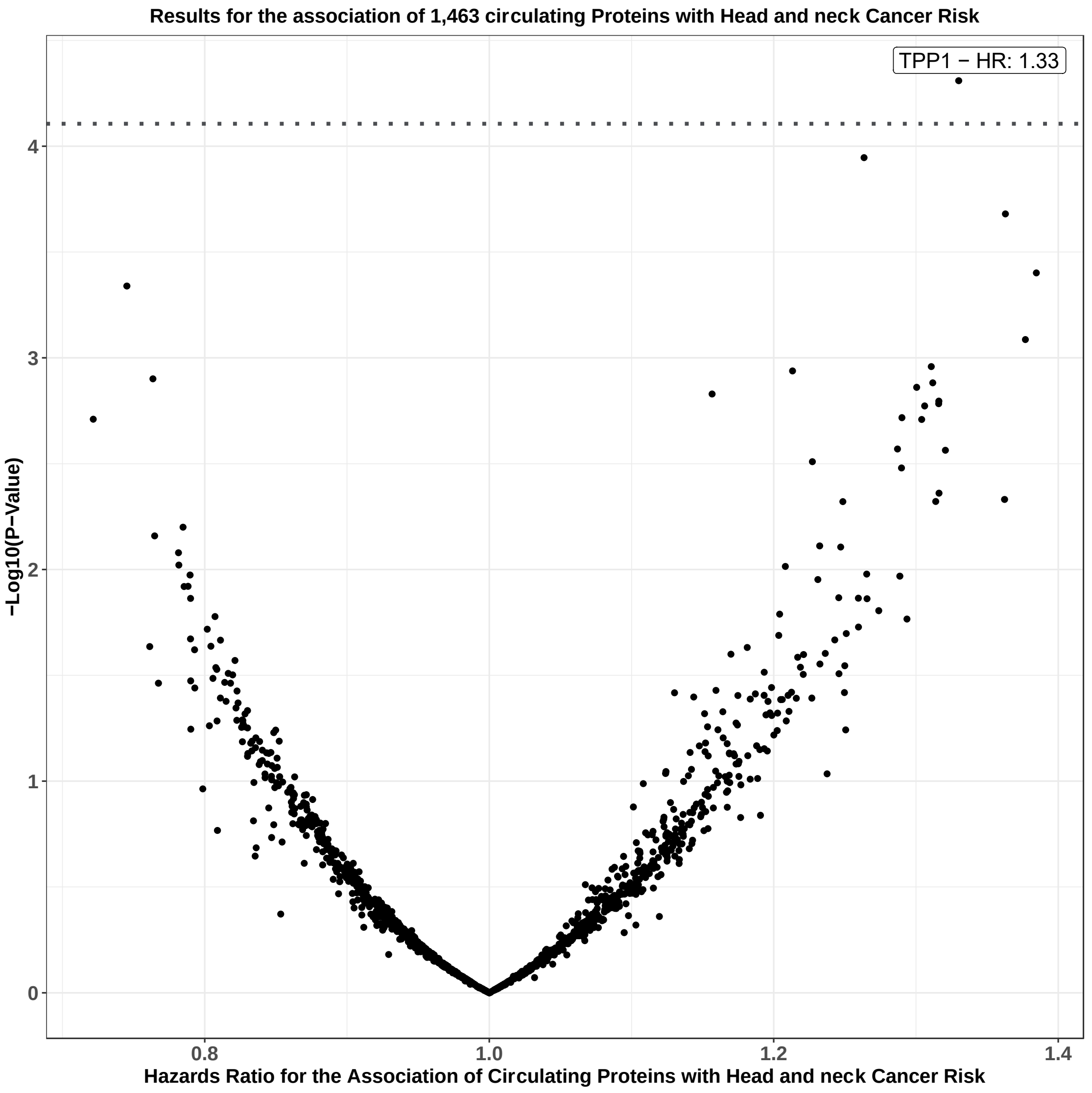

Supplementary Figure 7. Volcano plot for the prospective association of circulating proteins with risk of head and neck cancer

Volcano plots displaying the results from the prospective observational analyses of 1,463 proteins with risk of head and neck cancer. Hazard ratios per SD for cancer risk is plotted on the x-axis while –log10 p-values are plotted on the y-axis. Protein names and hazard ratios are labelled to highlight a selection of associations significant after correction for multiple testing ($p < 0.05/639$). Source data are provided as a Source Data file.

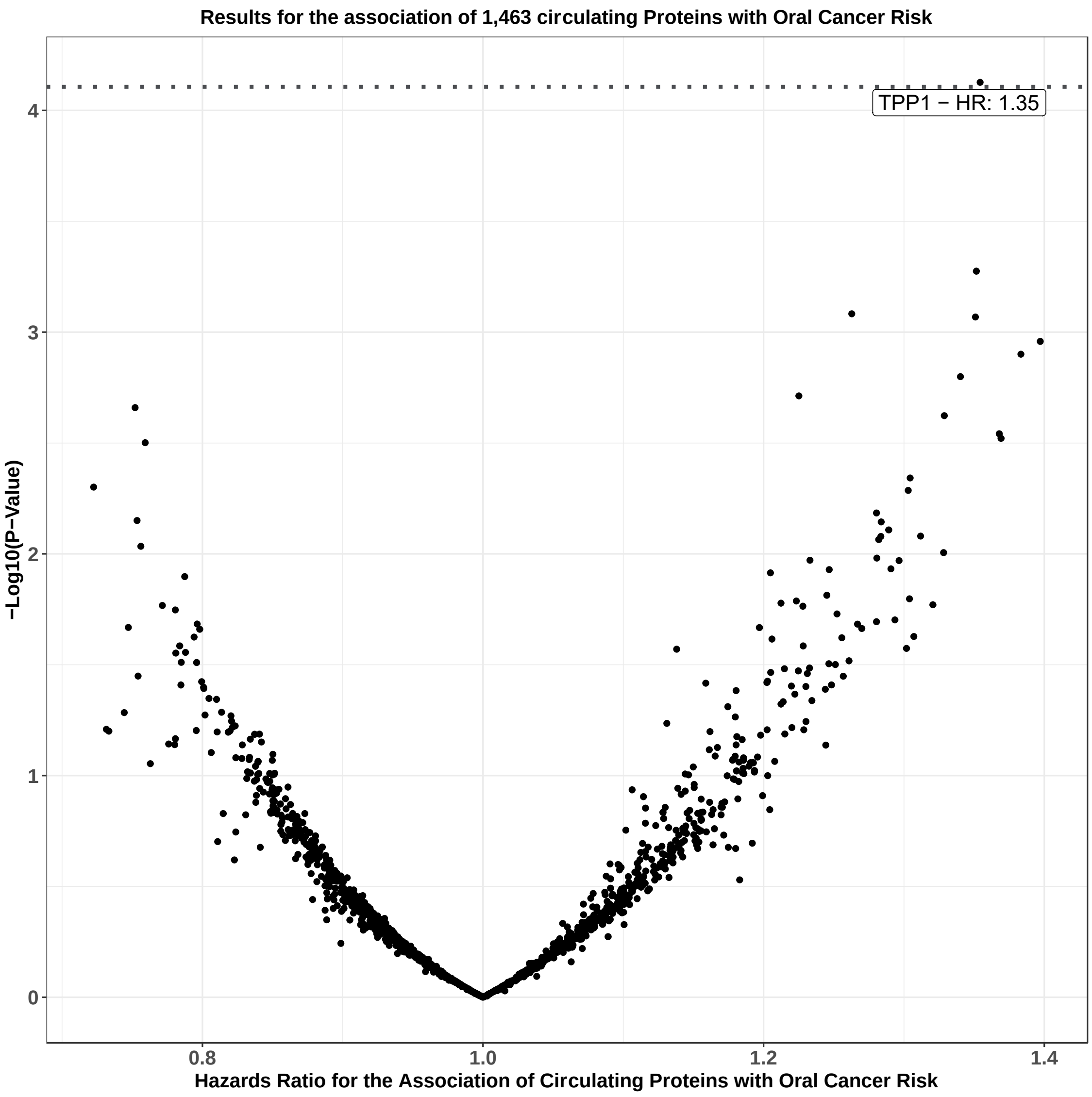

Supplementary Figure 8. Volcano plot for the prospective association of circulating proteins with risk of oral cancer

Volcano plots displaying the results from the prospective observational analyses of 1,463 proteins with risk of oral cancer. Hazard ratios per SD for cancer risk is plotted on the x-axis while –log10 p-values are plotted on the y-axis. Protein names and hazard ratios are labelled to highlight a selection of associations significant after correction for multiple testing ($p < 0.05/639$). Source data are provided as a Source Data file.

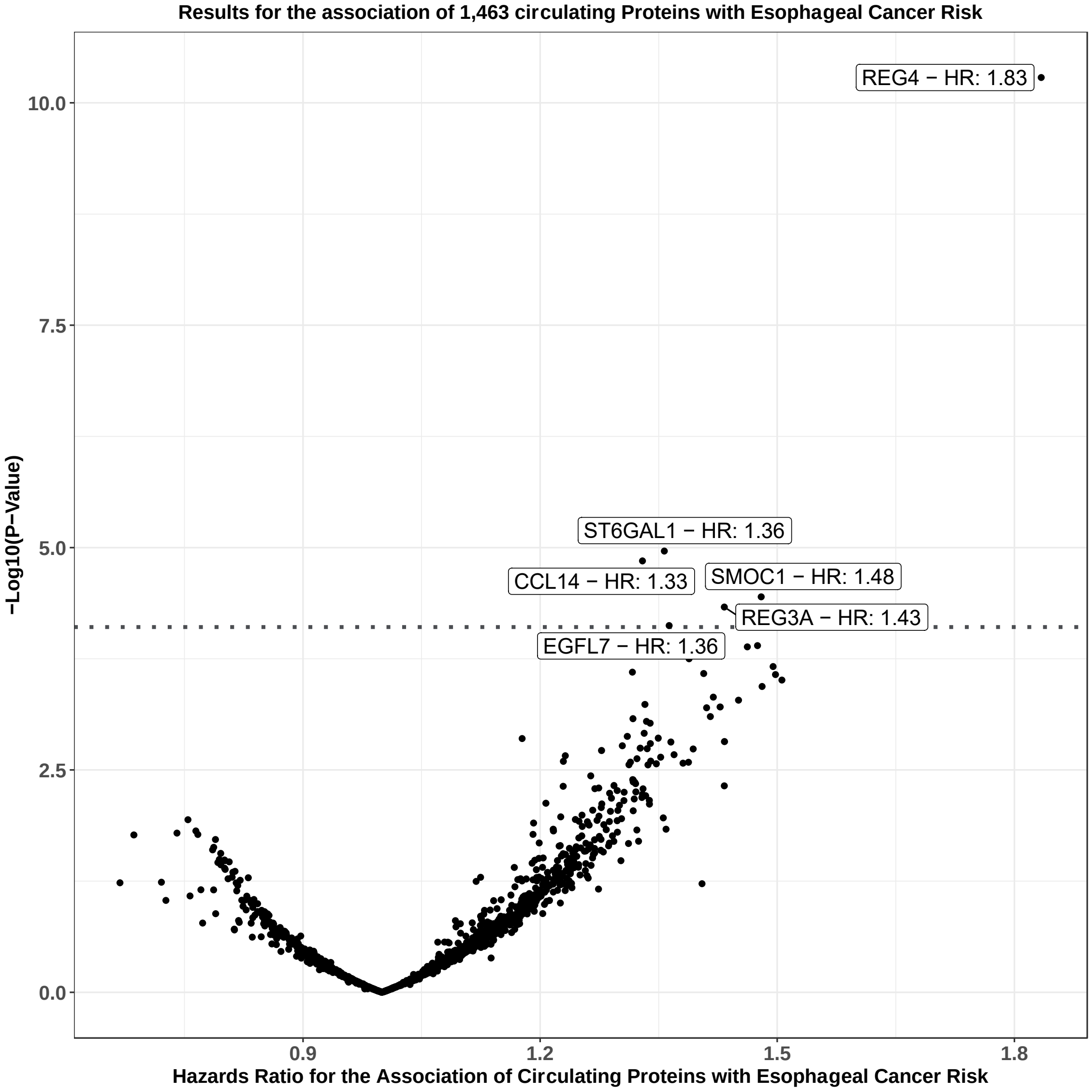

Supplementary Figure 9. Volcano plot for the prospective association of circulating proteins with risk of oesophagus cancer

Volcano plots displaying the results from the prospective observational analyses of 1,463 proteins with risk of oesophagus cancer. Hazard ratios per SD for cancer risk is plotted on the x-axis while –log10 p-values are plotted on the y-axis. Protein names and hazard ratios are labelled to highlight a selection of associations significant after correction for multiple testing ($p < 0.05/639$). Source data are provided as a Source Data file.

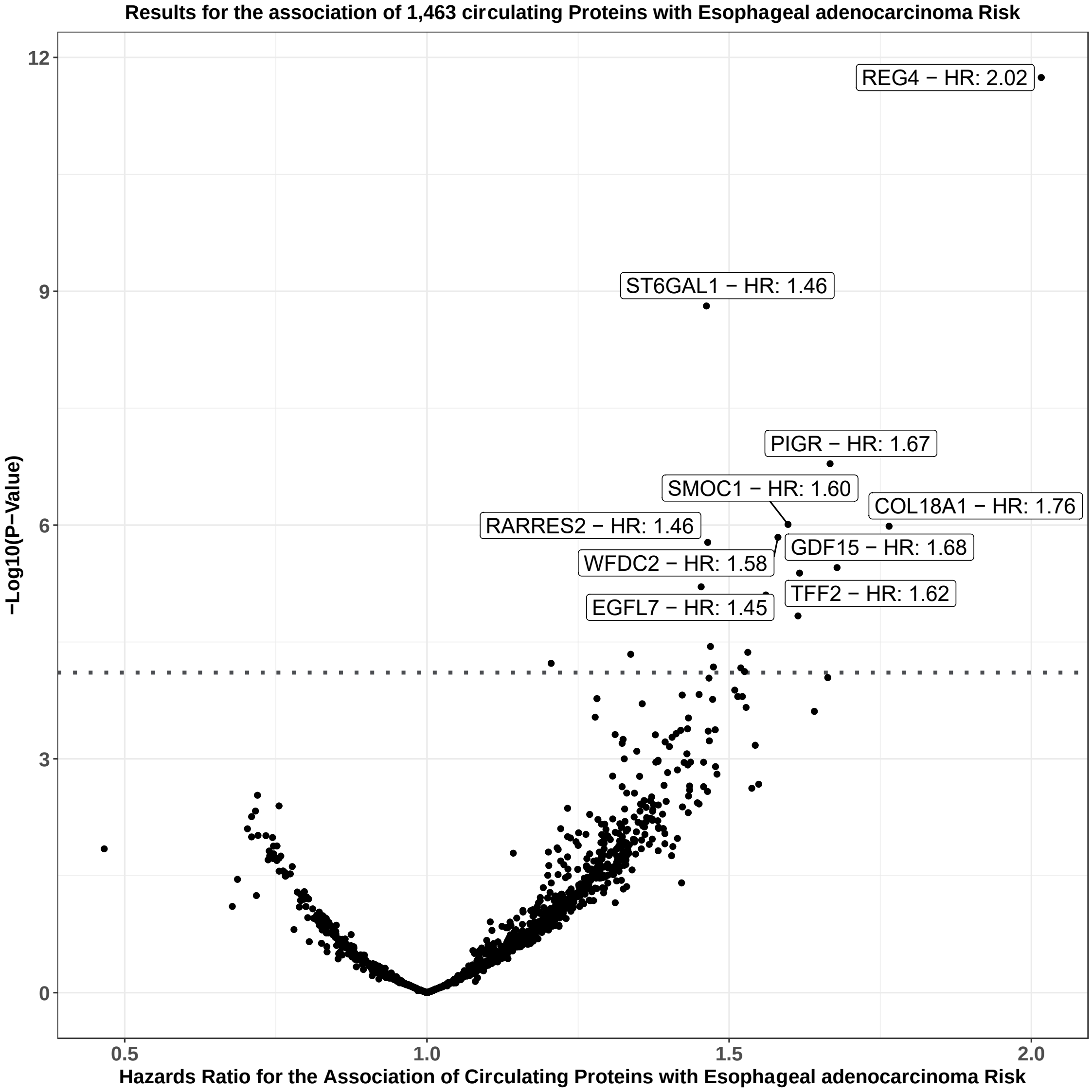

Supplementary Figure 10. Volcano plot for the prospective association of circulating proteins with risk of oesophageal adenocarcinoma

Volcano plots displaying the results from the prospective observational analyses of 1,463 proteins with risk of oesophageal adenocarcinoma. Hazard ratios per SD for cancer risk is plotted on the x-axis while –log10 p-values are plotted on the y-axis. Protein names and hazard ratios are labelled to highlight a selection of associations significant after correction for multiple testing ($p < 0.05/639$). Source data are provided as a Source Data file.

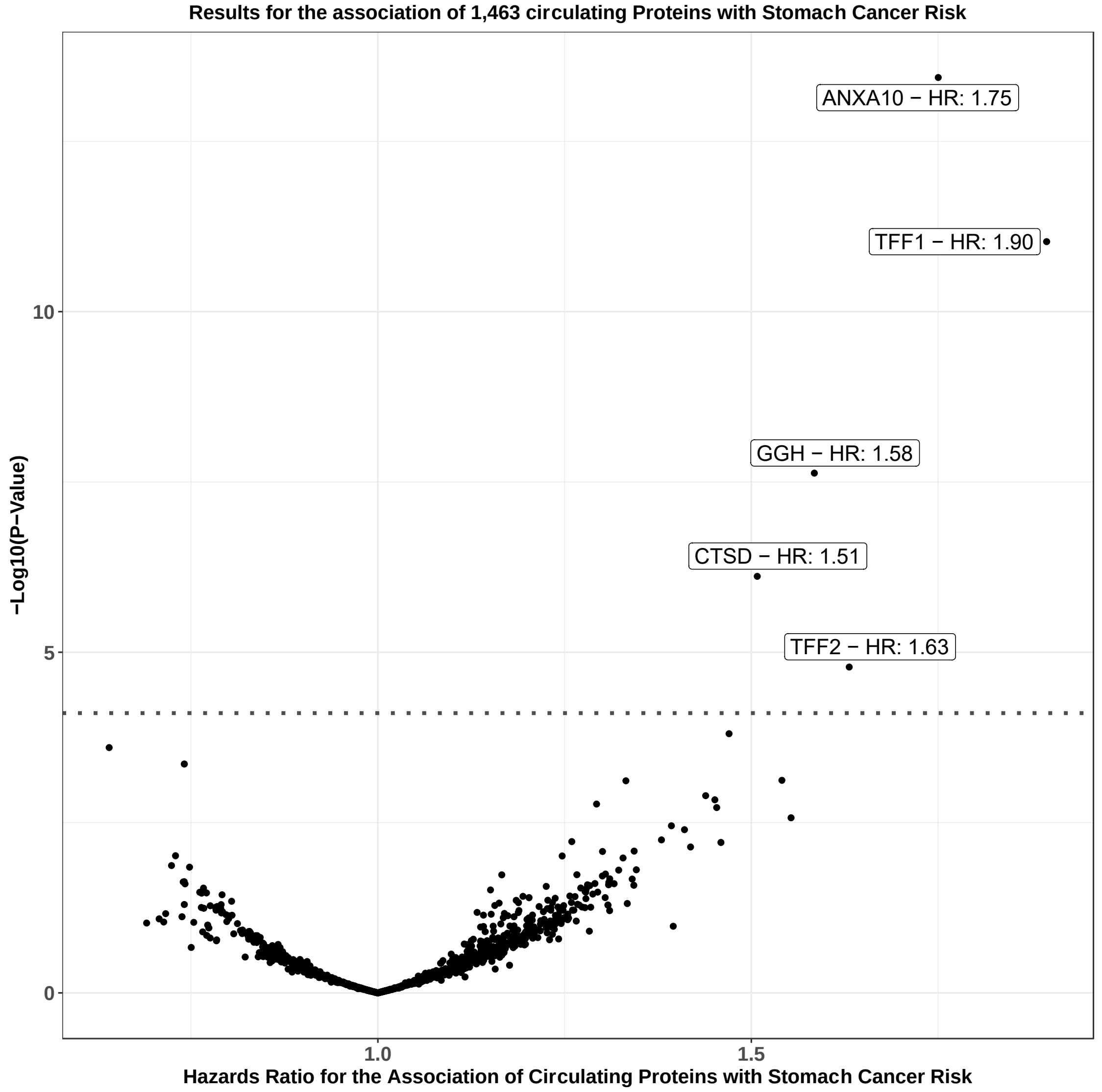

Supplementary Figure 11. Volcano plot for the prospective association of circulating proteins with risk of stomach cancer

Volcano plots displaying the results from the prospective observational analyses of 1,463 proteins with risk of stomach cancer. Hazard ratios per SD for cancer risk is plotted on the x-axis while –log10 p-values are plotted on the y-axis. Protein names and hazard ratios are labelled to highlight a selection of associations significant after correction for multiple testing ($p < 0.05/639$). Source data are provided as a Source Data file.

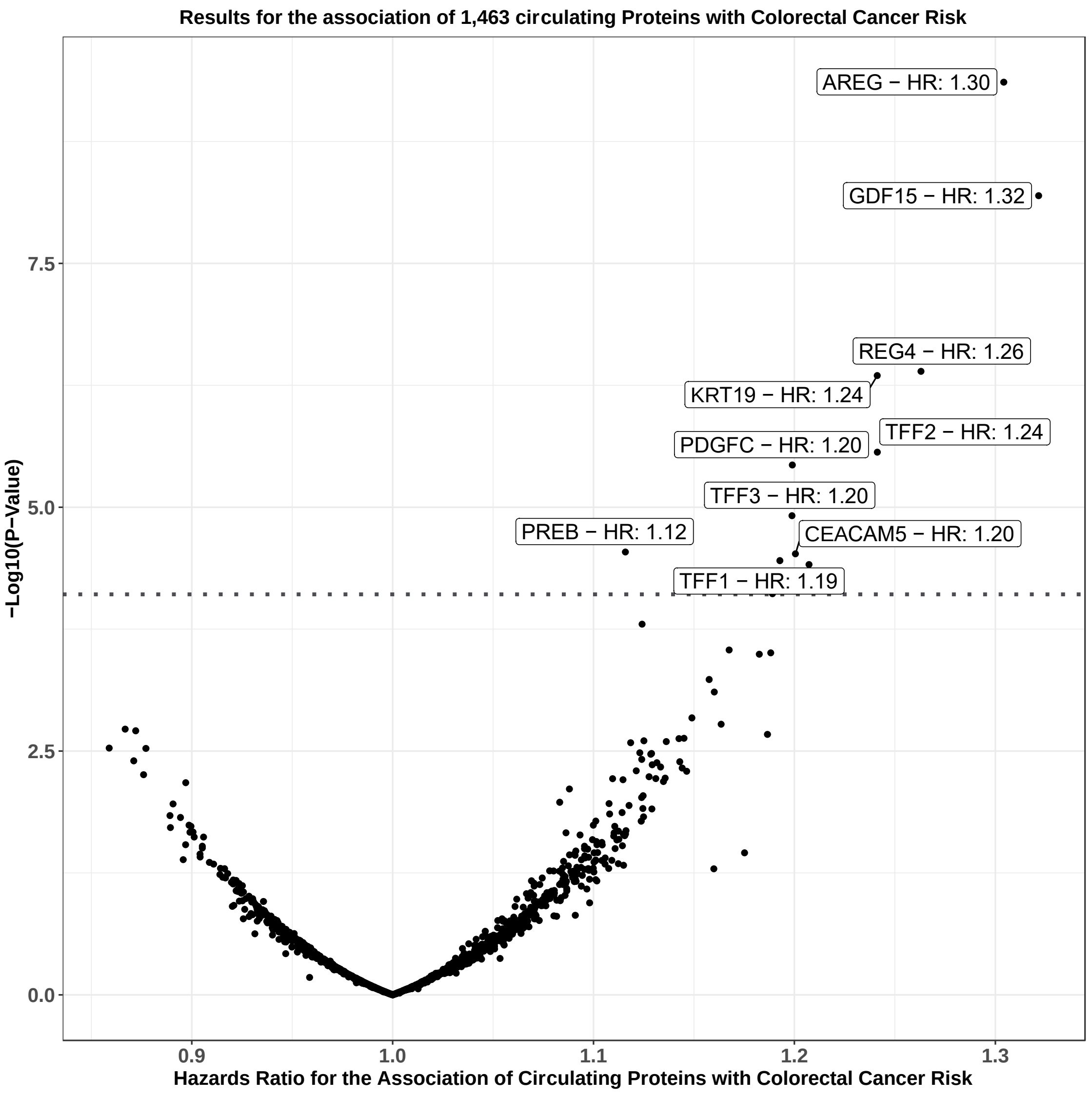

Supplementary Figure 12. Volcano plot for the prospective association of circulating proteins with risk of colorectum cancer

Volcano plots displaying the results from the prospective observational analyses of 1,463 proteins with risk of colorectum cancer. Hazard ratios per SD for cancer risk is plotted on the x-axis while –log10 p-values are plotted on the y-axis. Protein names and hazard ratios are labelled to highlight a selection of associations significant after correction for multiple testing (p < 0.05/639). Source data are provided as a Source Data file.

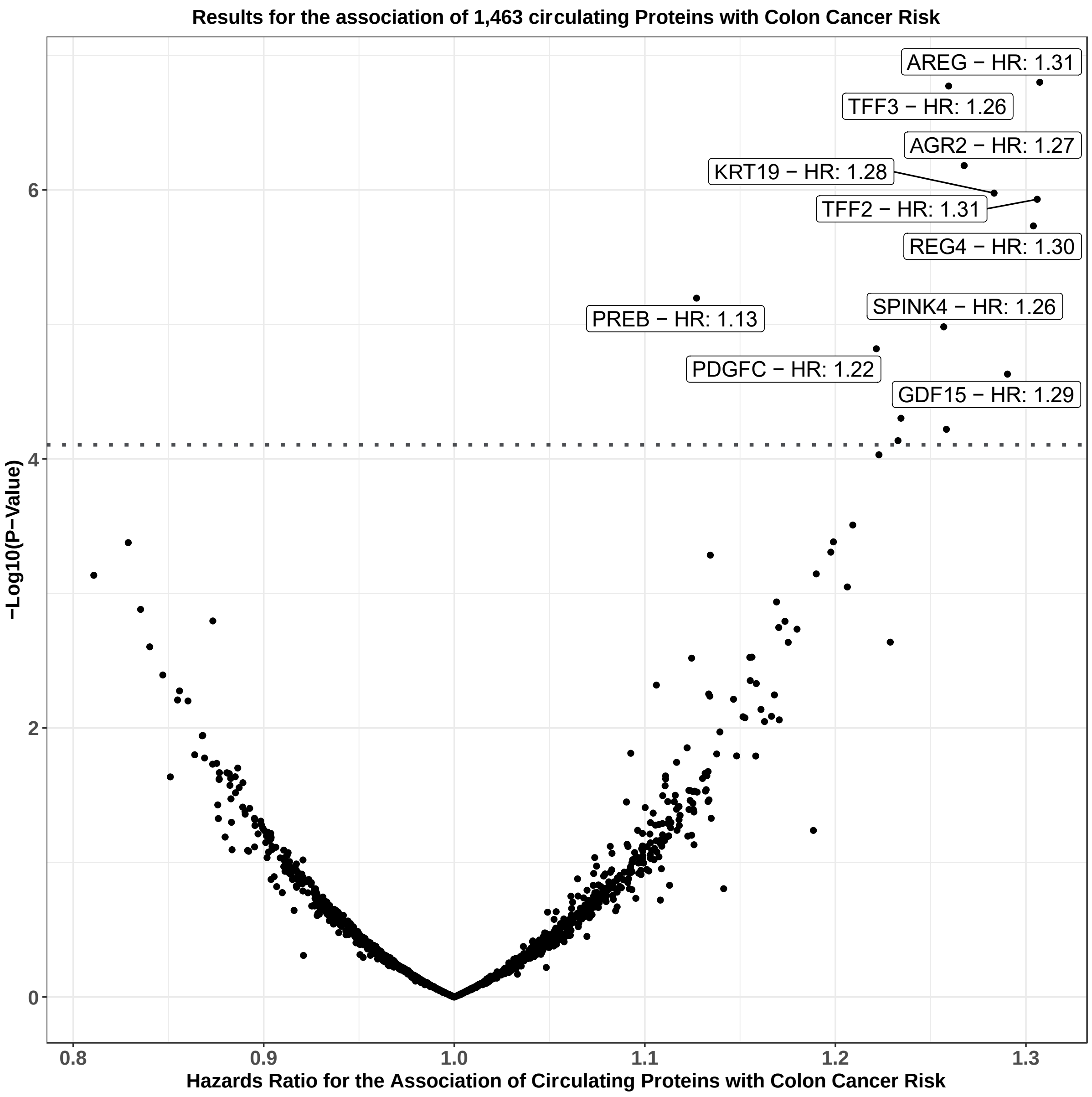

Supplementary Figure 13. Volcano plot for the prospective association of circulating proteins with risk of colon cancer

Volcano plots displaying the results from the prospective observational analyses of 1,463 proteins with risk of colon cancer. Hazard ratios per SD for cancer risk is plotted on the x-axis while –log10 p-values are plotted on the y-axis. Protein names and hazard ratios are labelled to highlight a selection of associations significant after correction for multiple testing (p < 0.05/639). Source data are provided as a Source Data file.

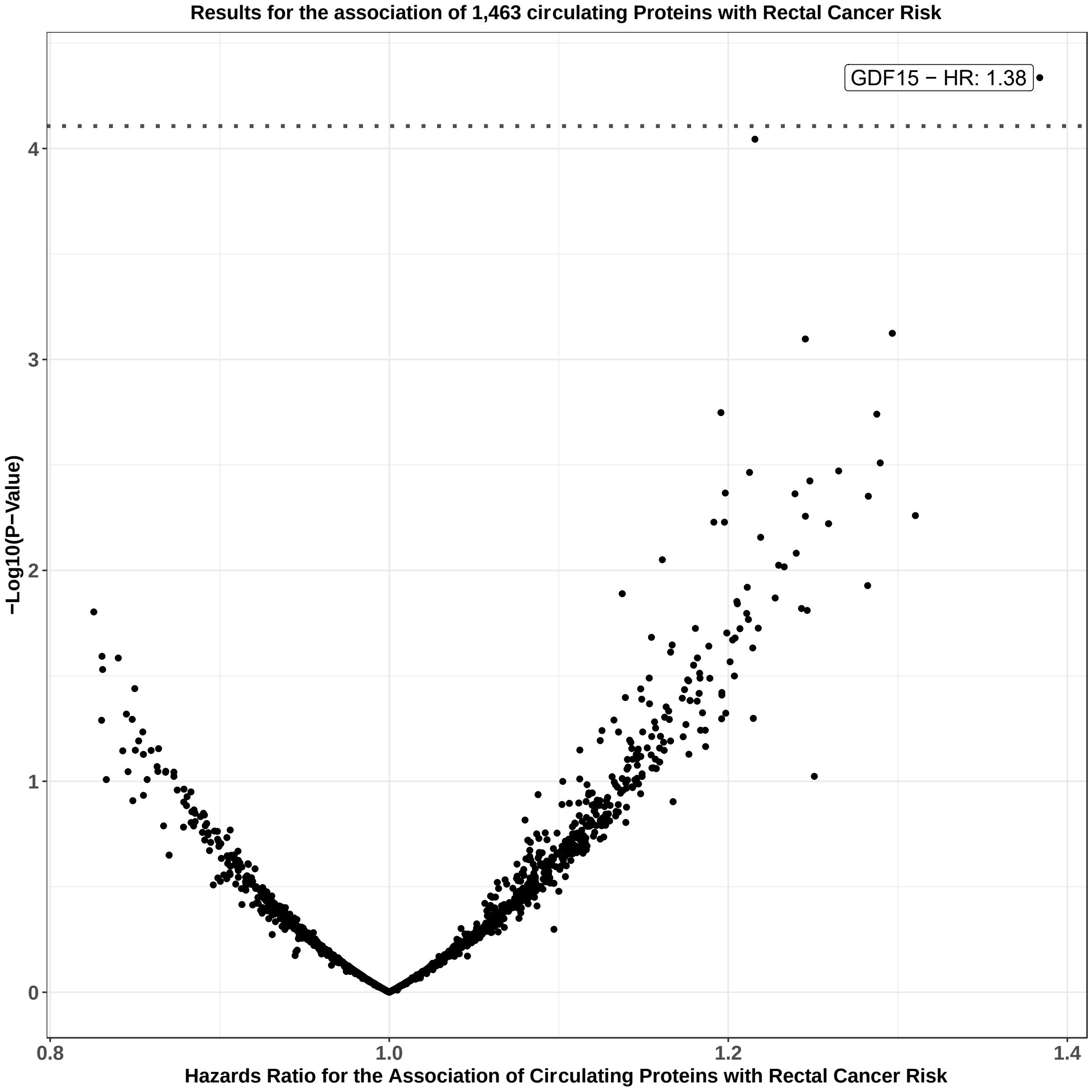

Supplementary Figure 14. Volcano plot for the prospective association of circulating proteins with risk of rectal cancer

Volcano plots displaying the results from the prospective observational analyses of 1,463 proteins with risk of rectal cancer. Hazard ratios per SD for cancer risk is plotted on the x-axis while –log10 p-values are plotted on the y-axis. Protein names and hazard ratios are labelled to highlight a selection of associations significant after correction for multiple testing ($p < 0.05/639$). Source data are provided as a Source Data file.

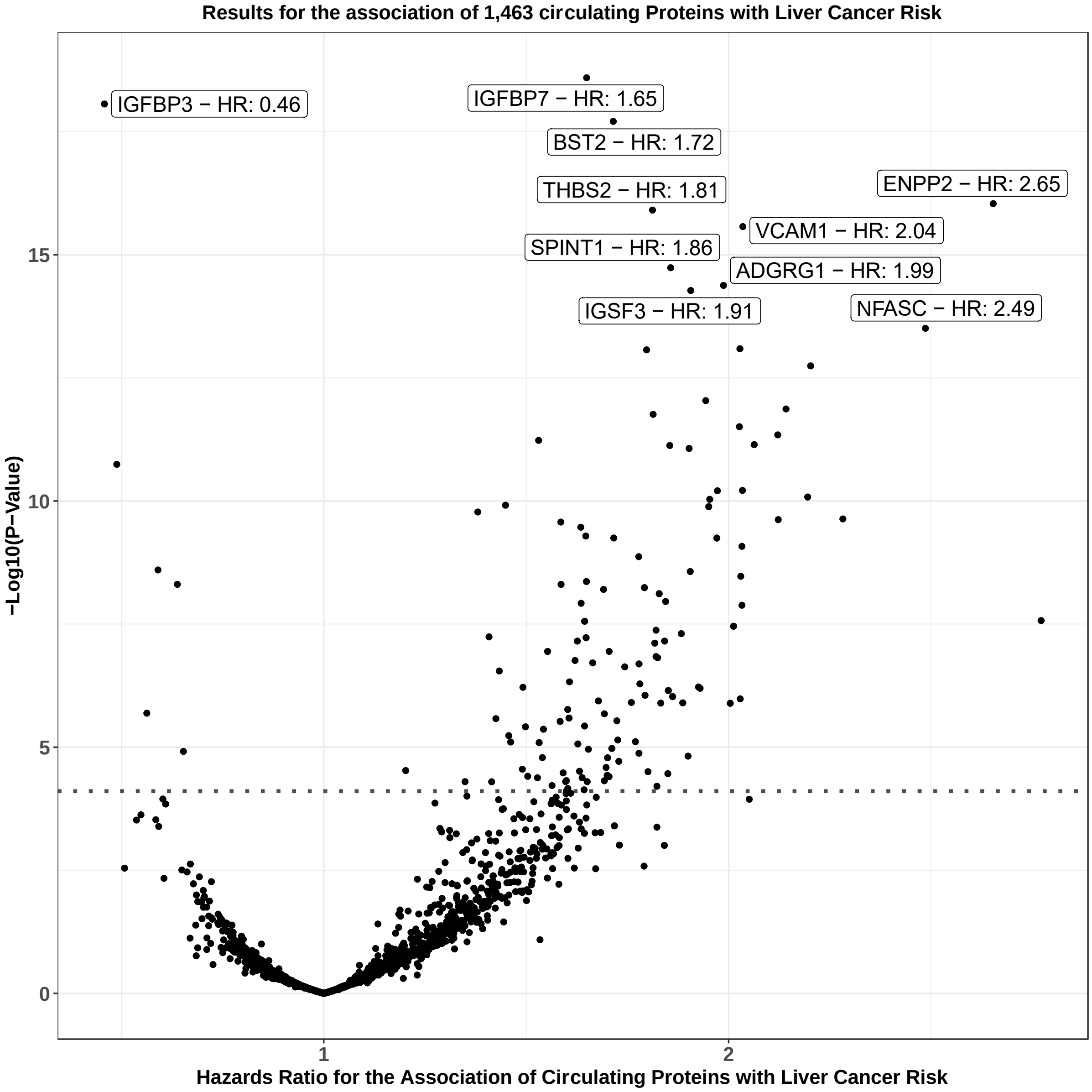

Supplementary Figure 15. Volcano plot for the prospective association of circulating proteins with risk of liver cancer

Volcano plots displaying the results from the prospective observational analyses of 1,463 proteins with risk of liver cancer. Hazard ratios per SD for cancer risk is plotted on the x-axis while –log10 p-values are plotted on the y-axis. Protein names and hazard ratios are labelled to highlight a selection of associations significant after correction for multiple testing ($p < 0.05/639$). Source data are provided as a Source Data file.

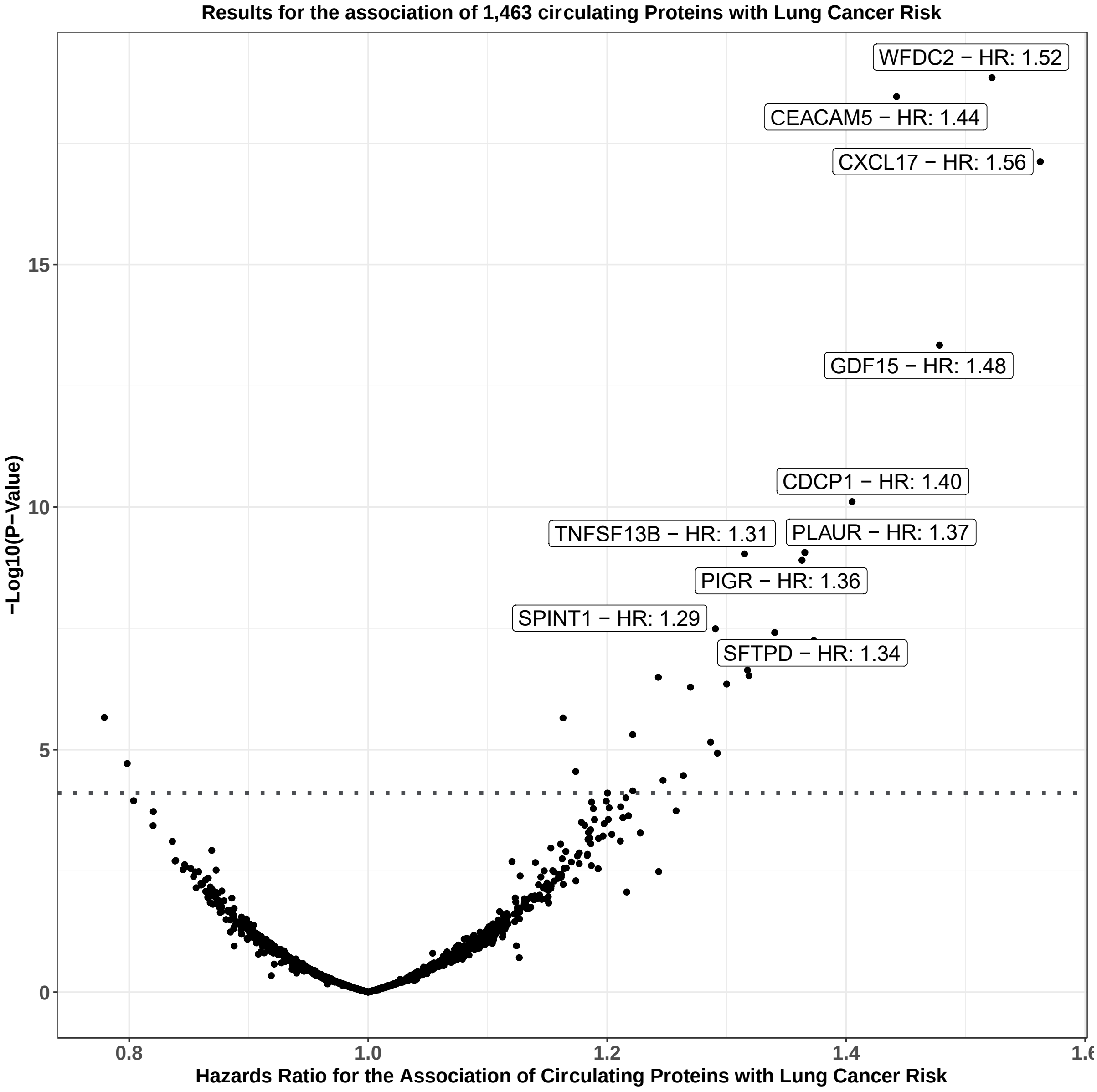

Supplementary Figure 16. Volcano plot for the prospective association of circulating proteins with risk of lung cancer

Volcano plots displaying the results from the prospective observational analyses of 1,463 proteins with risk of lung cancer. Hazard ratios per SD for cancer risk is plotted on the x-axis while –log10 p-values are plotted on the y-axis. Protein names and hazard ratios are labelled to highlight a selection of associations significant after correction for multiple testing ($p < 0.05/639$). Source data are provided as a Source Data file.

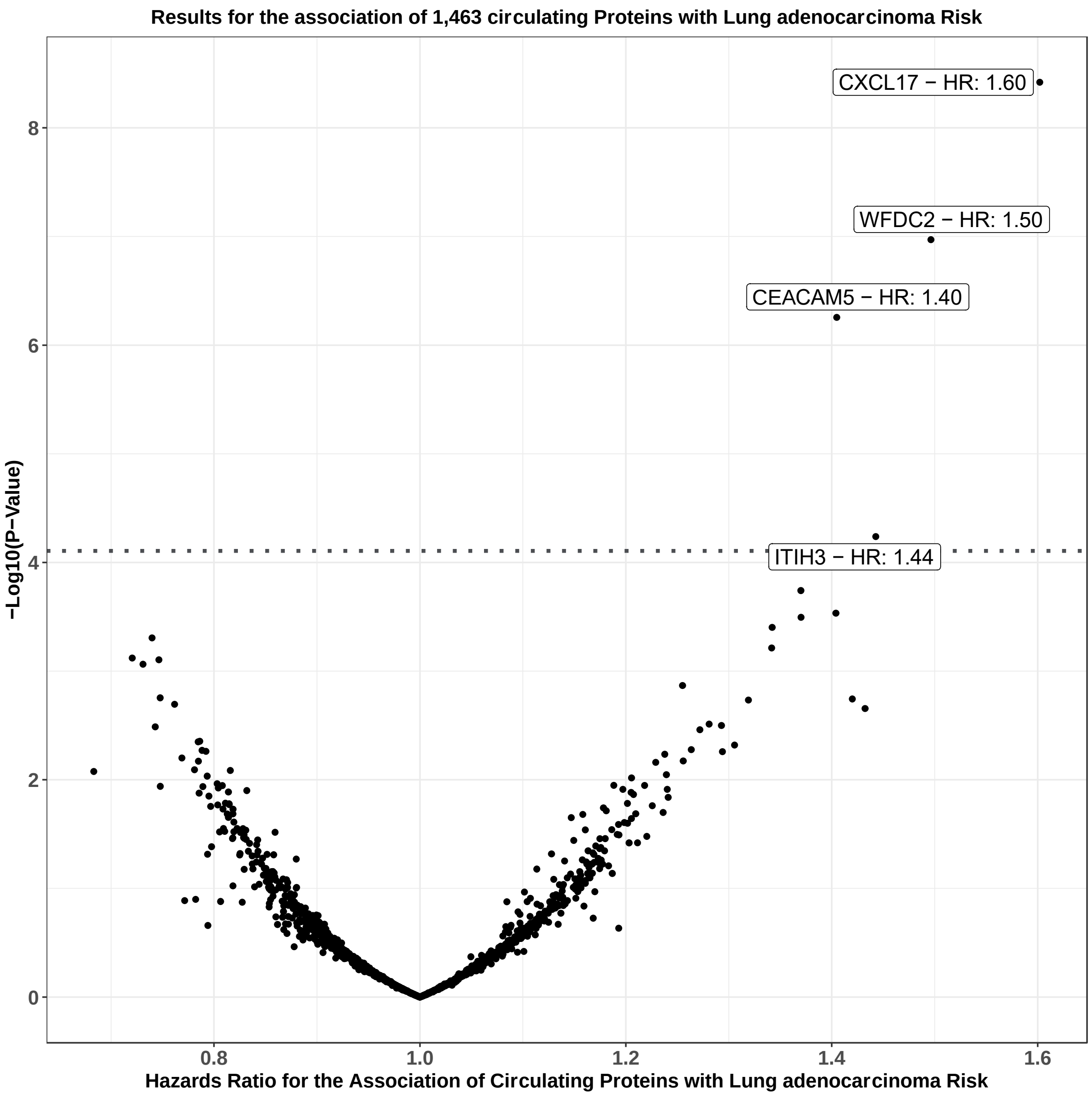

Supplementary Figure 17. Volcano plot for the prospective association of circulating proteins with risk of lung adenocarcinoma

Volcano plots displaying the results from the prospective observational analyses of 1,463 proteins with risk of lung adenocarcinoma. Hazard ratios per SD for cancer risk is plotted on the x-axis while –log10 p-values are plotted on the y-axis. Protein names and hazard ratios are labelled to highlight a selection of associations significant after correction for multiple testing ($p < 0.05/639$). Source data are provided as a Source Data file.

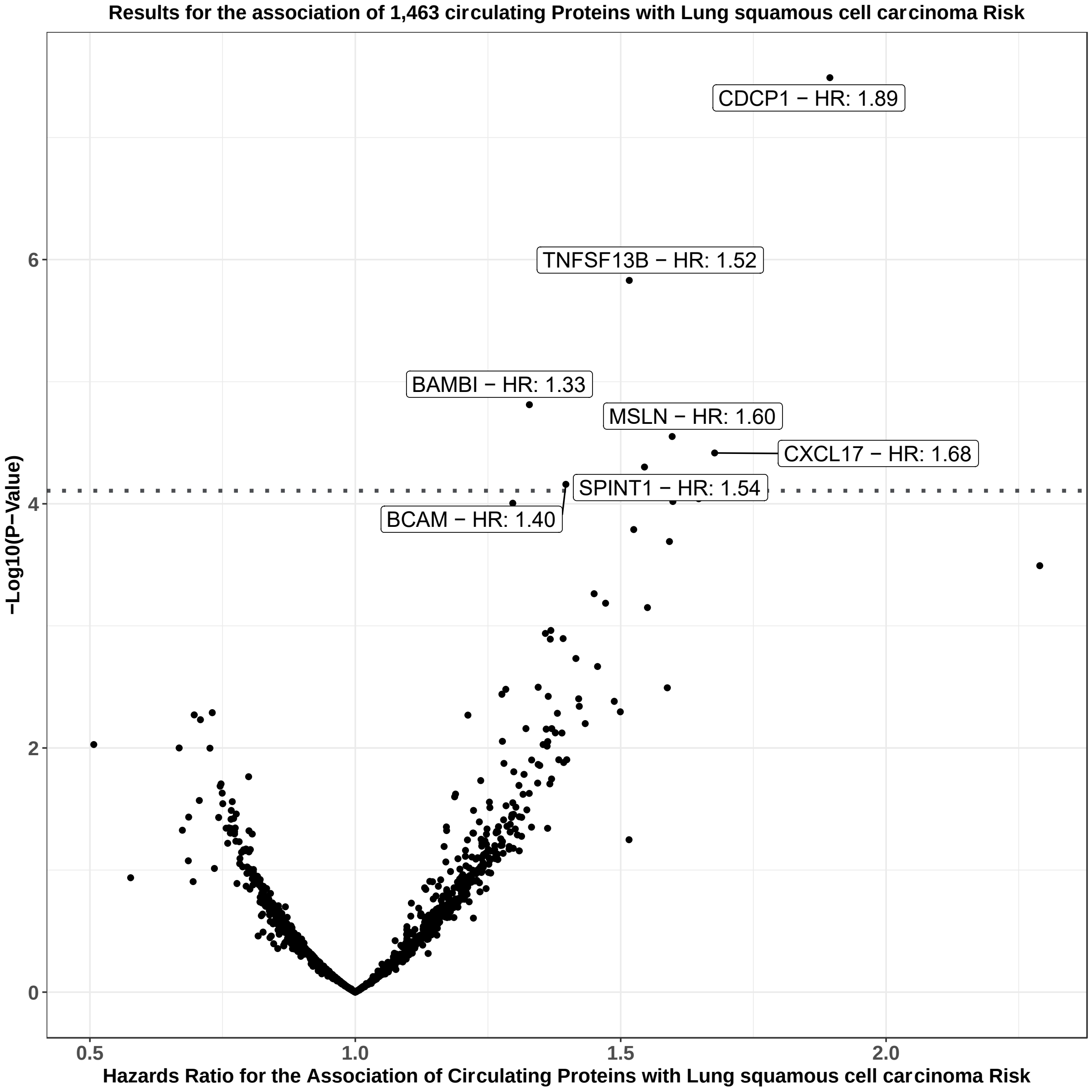

Supplementary Figure 18. Volcano plot for the prospective association of circulating proteins with risk of lung squamous cell carcinoma

Volcano plots displaying the results from the prospective observational analyses of 1,463 proteins with risk of lung squamous cell carcinoma. Hazard ratios per SD for cancer risk is plotted on the x-axis while –log10 p-values are plotted on the y-axis. Protein names and hazard ratios are labelled to highlight a selection of associations significant after correction for multiple testing ($p < 0.05/639$). Source data are provided as a Source Data file.

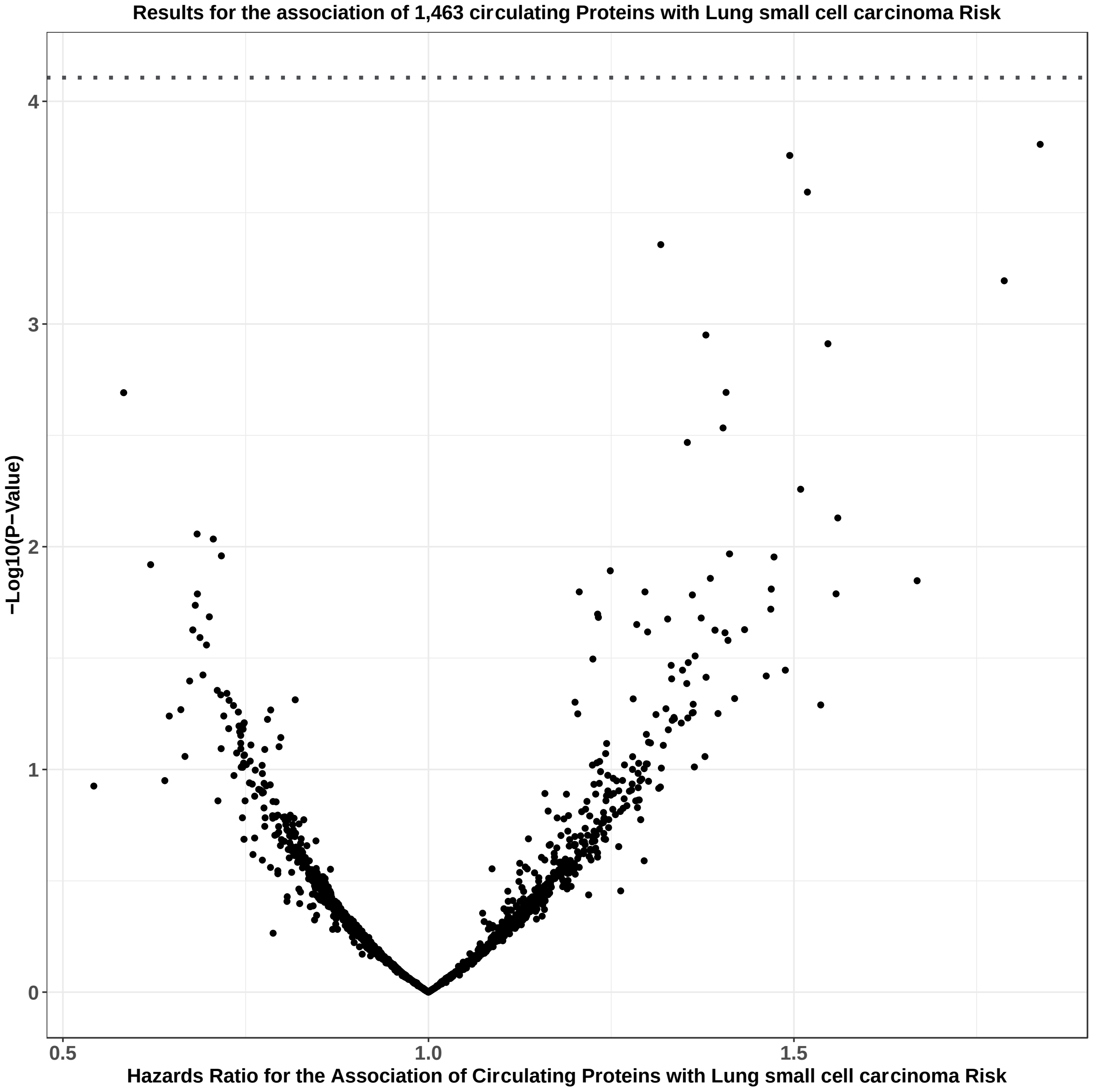

Supplementary Figure 19. Volcano plot for the prospective association of circulating proteins with risk of lung small cell carcinoma

Volcano plots displaying the results from the prospective observational analyses of 1,463 proteins with risk of lung small cell carcinoma. Hazard ratios per SD for cancer risk is plotted on the x-axis while –log10 p-values are plotted on the y-axis. Protein names and hazard ratios are labelled to highlight a selection of associations significant after correction for multiple testing ($p < 0.05/639$). Source data are provided as a Source Data file.

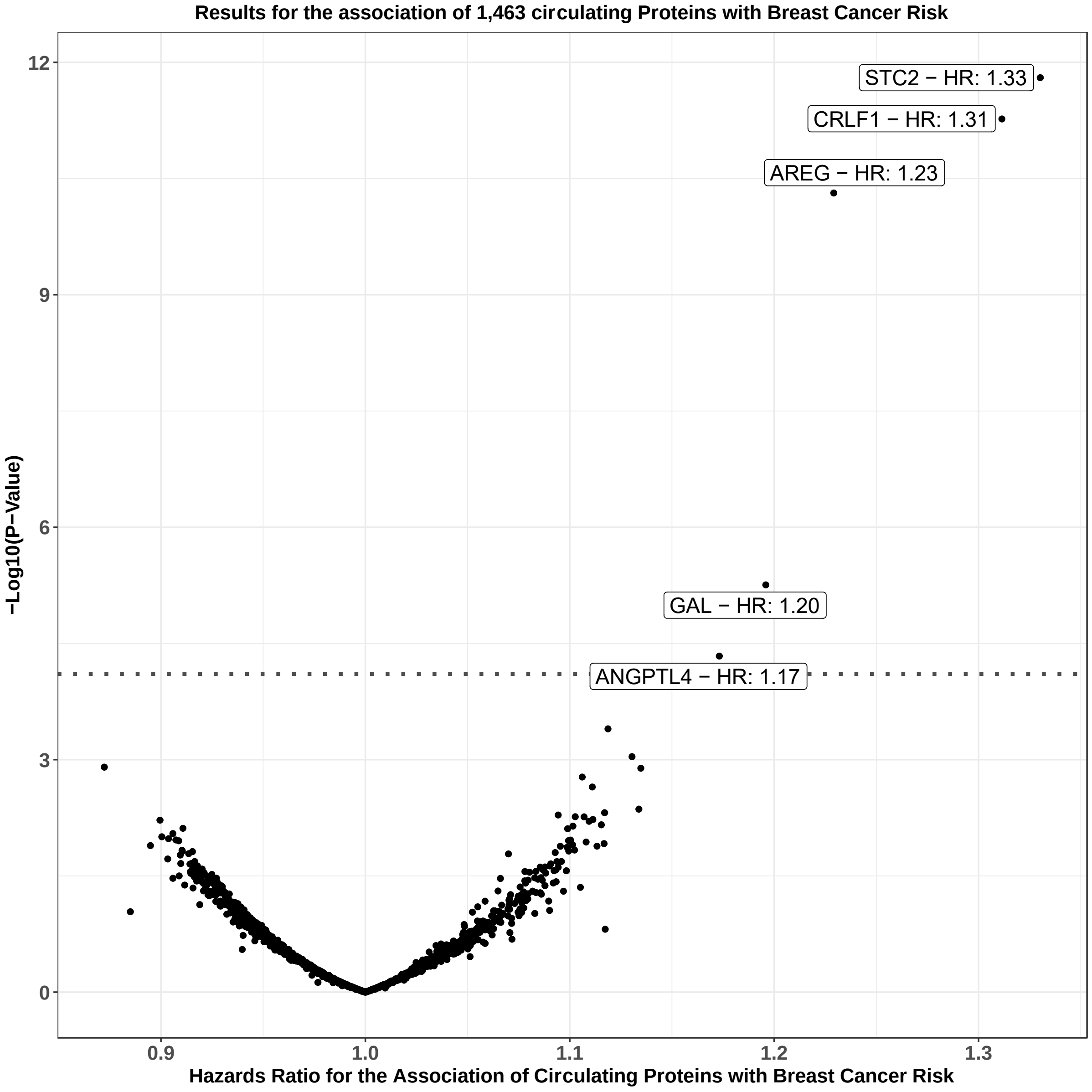

Supplementary Figure 20. Volcano plot for the prospective association of circulating proteins with risk of breast cancer

Volcano plots displaying the results from the prospective observational analyses of 1,463 proteins with risk of breast cancer. Hazard ratios per SD for cancer risk is plotted on the x-axis while –log10 p-values are plotted on the y-axis. Protein names and hazard ratios are labelled to highlight a selection of associations significant after correction for multiple testing ($p < 0.05/639$). Source data are provided as a Source Data file.

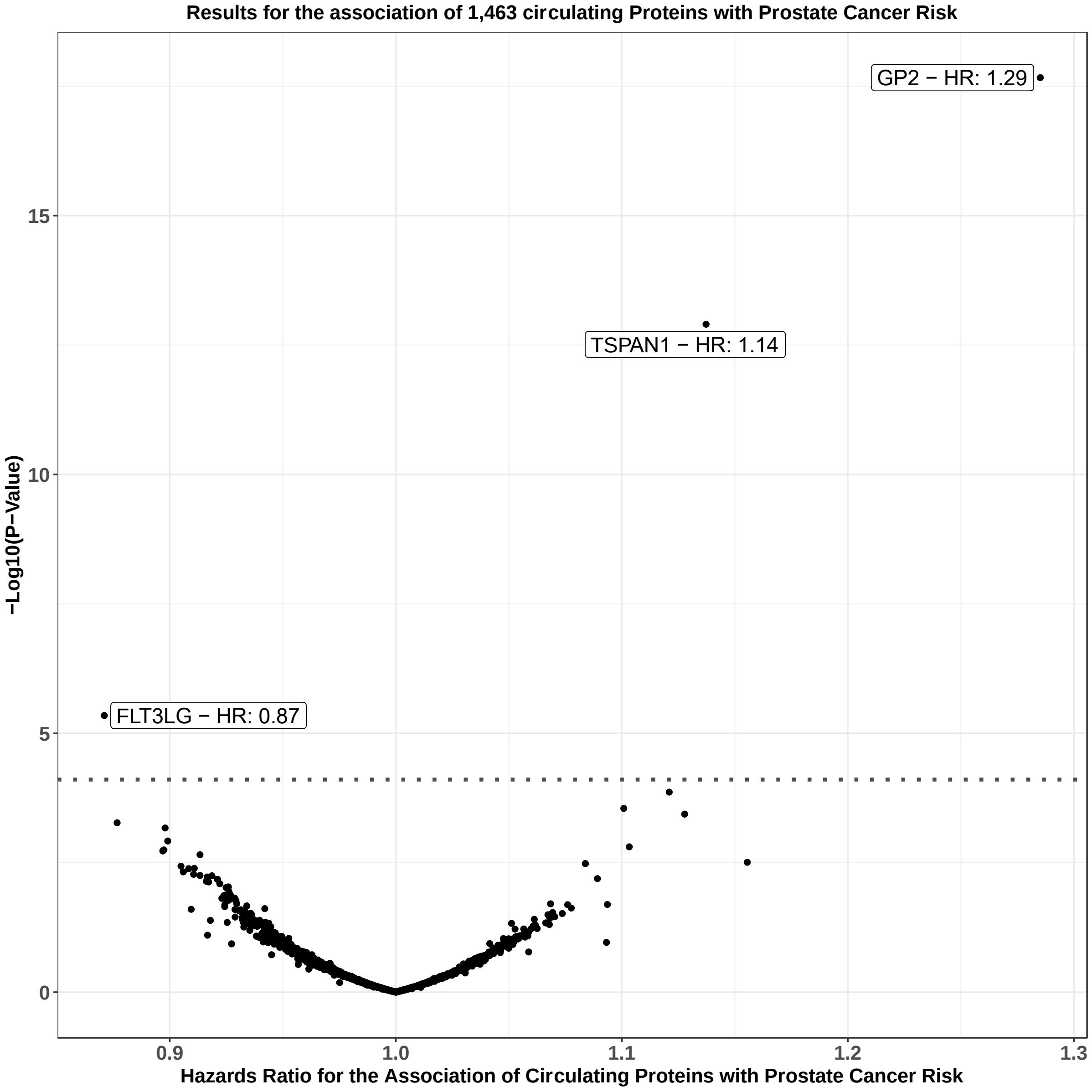

Supplementary Figure 21. Volcano plot for the prospective association of circulating proteins with risk of prostate cancer

Volcano plots displaying the results from the prospective observational analyses of 1,463 proteins with risk of prostate cancer. Hazard ratios per SD for cancer risk is plotted on the x-axis while –log10 p-values are plotted on the y-axis. Protein names and hazard ratios are labelled to highlight a selection of associations significant after correction for multiple testing ($p < 0.05/639$). Source data are provided as a Source Data file.

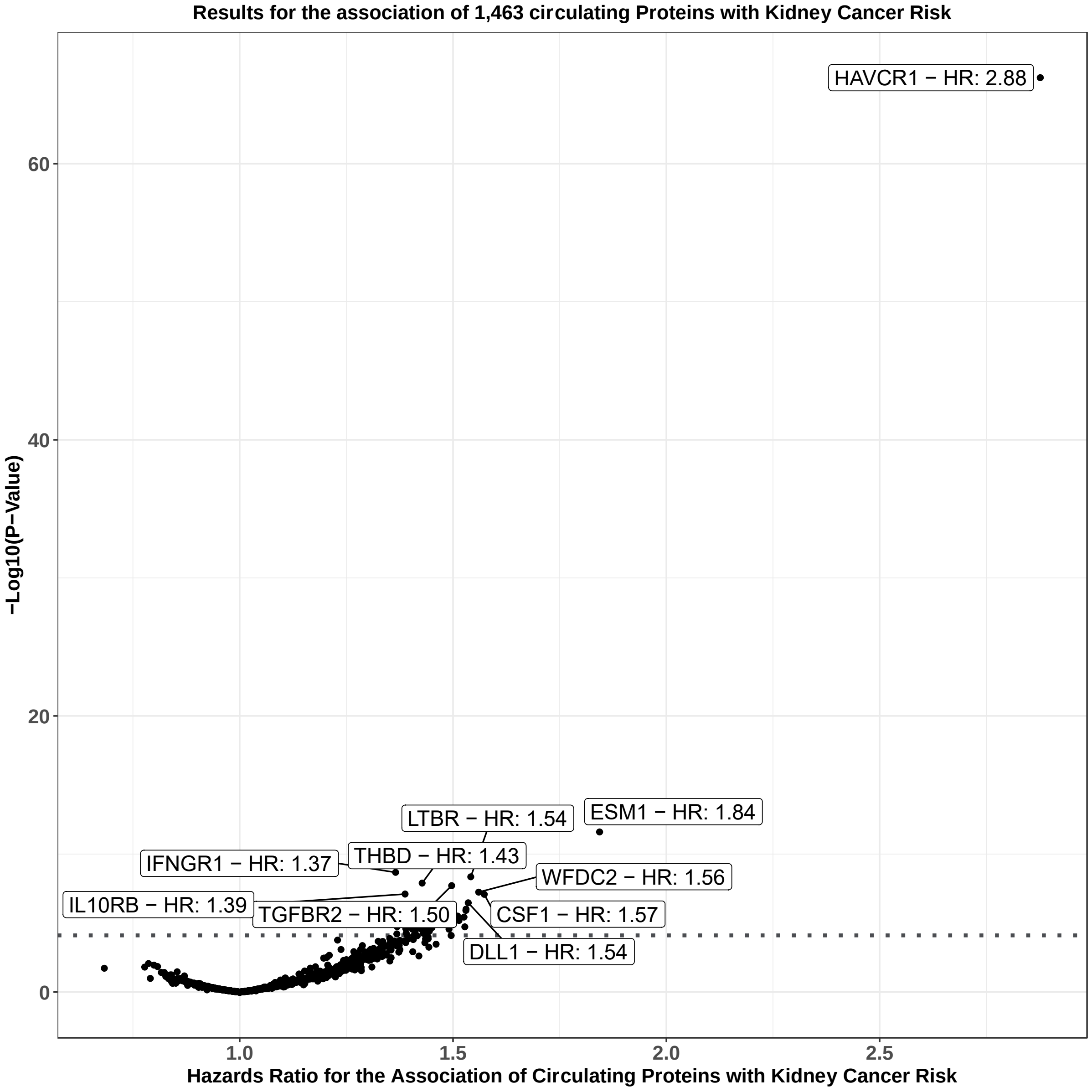

Supplementary Figure 22. Volcano plot for the prospective association of circulating proteins with risk of kidney cancer

Volcano plots displaying the results from the prospective observational analyses of 1,463 proteins with risk of kidney cancer. Hazard ratios per SD for cancer risk is plotted on the x-axis while –log10 p-values are plotted on the y-axis. Protein names and hazard ratios are labelled to highlight a selection of associations significant after correction for multiple testing ($p < 0.05/639$). Source data are provided as a Source Data file.

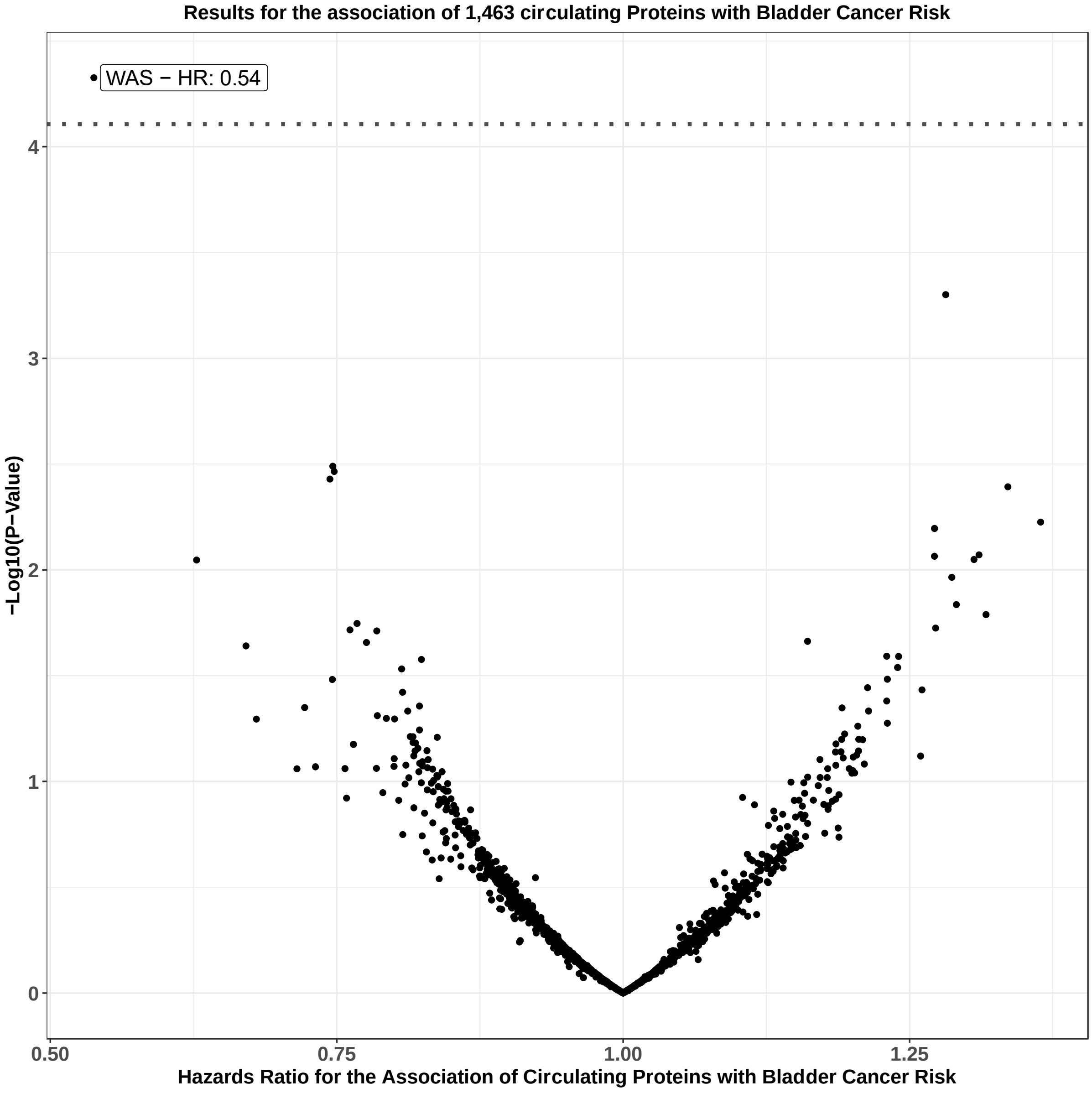

Supplementary Figure 23. Volcano plot for the prospective association of circulating proteins with risk of bladder cancer

Volcano plots displaying the results from the prospective observational analyses of 1,463 proteins with risk of bladder cancer. Hazard ratios per SD for cancer risk is plotted on the x-axis while –log10 p-values are plotted on the y-axis. Protein names and hazard ratios are labelled to highlight a selection of associations significant after correction for multiple testing ($p < 0.05/639$). Source data are provided as a Source Data file.

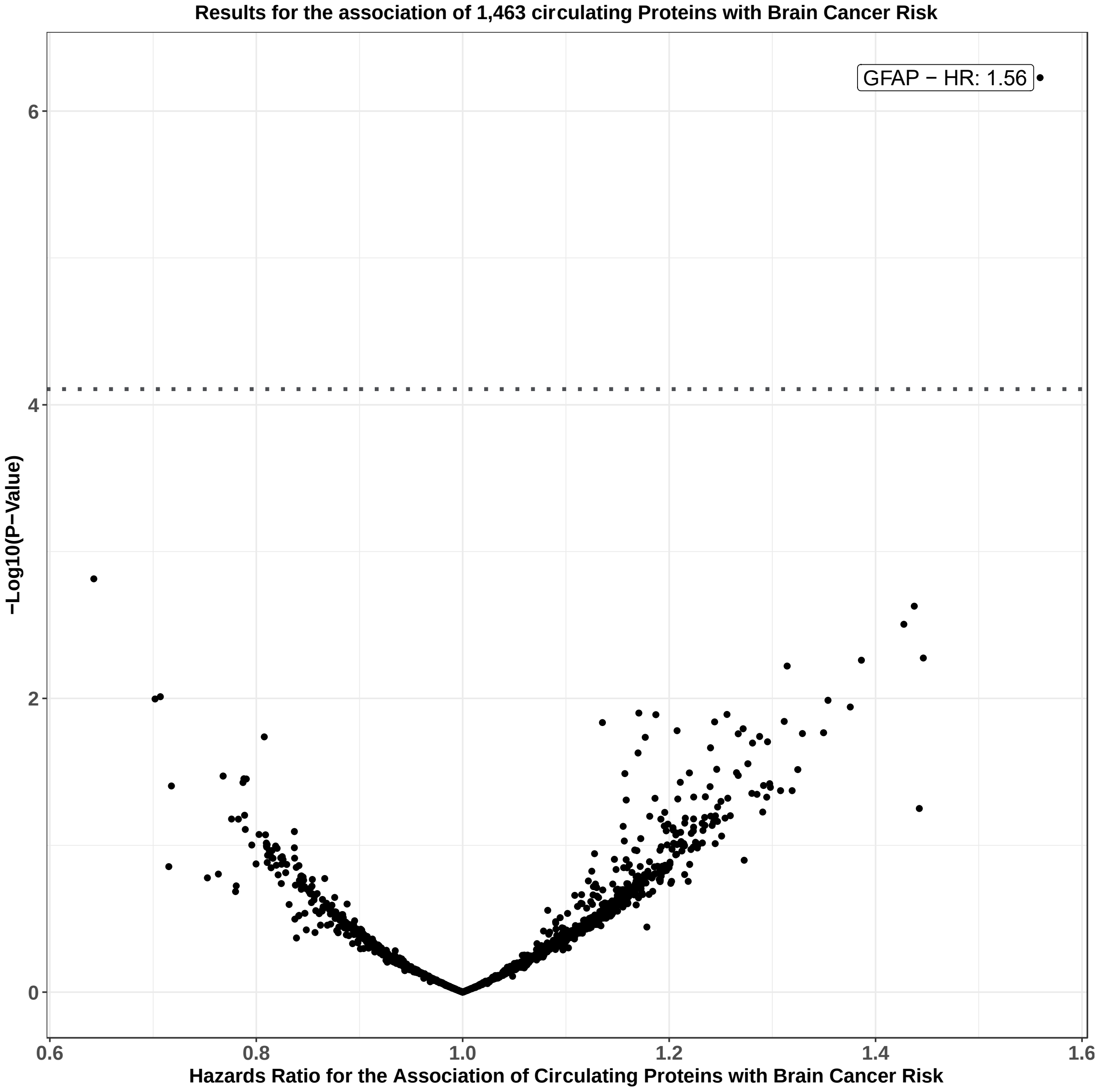

Supplementary Figure 24. Volcano plot for the prospective association of circulating proteins with risk of brain cancer

Volcano plots displaying the results from the prospective observational analyses of 1,463 proteins with risk of brain cancer. Hazard ratios per SD for cancer risk is plotted on the x-axis while –log10 p-values are plotted on the y-axis. Protein names and hazard ratios are labelled to highlight a selection of associations significant after correction for multiple testing ($p < 0.05/639$). Source data are provided as a Source Data file.

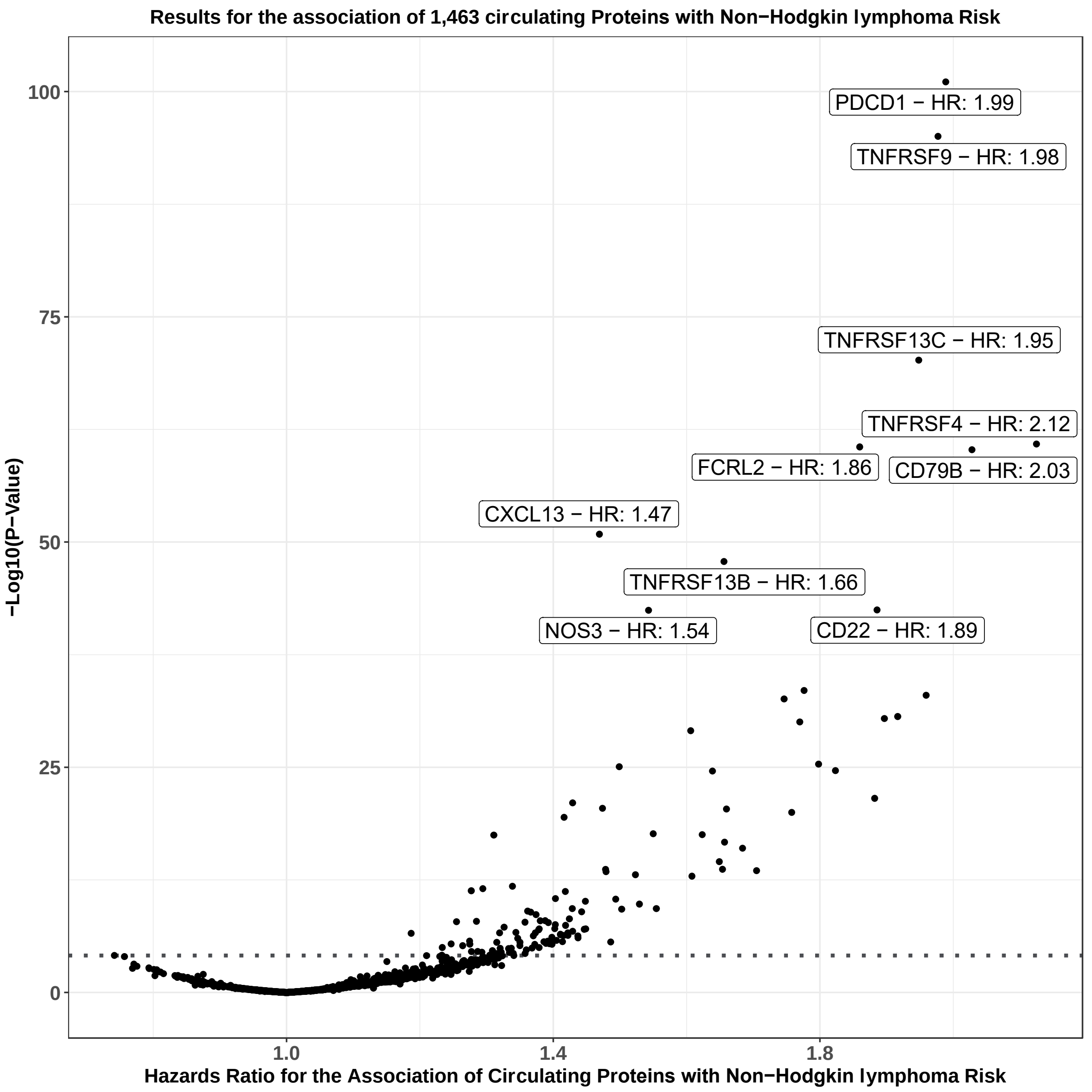

Supplementary Figure 25. Volcano plot for the prospective association of circulating proteins with risk of non-Hodgkin lymphoma

Volcano plots displaying the results from the prospective observational analyses of 1,463 proteins with risk of non-Hodgkin lymphoma. Hazard ratios per SD for cancer risk is plotted on the x-axis while –log10 p-values are plotted on the y-axis. Protein names and hazard ratios are labelled to highlight a selection of associations significant after correction for multiple testing (p < 0.05/639). Source data are provided as a Source Data file.

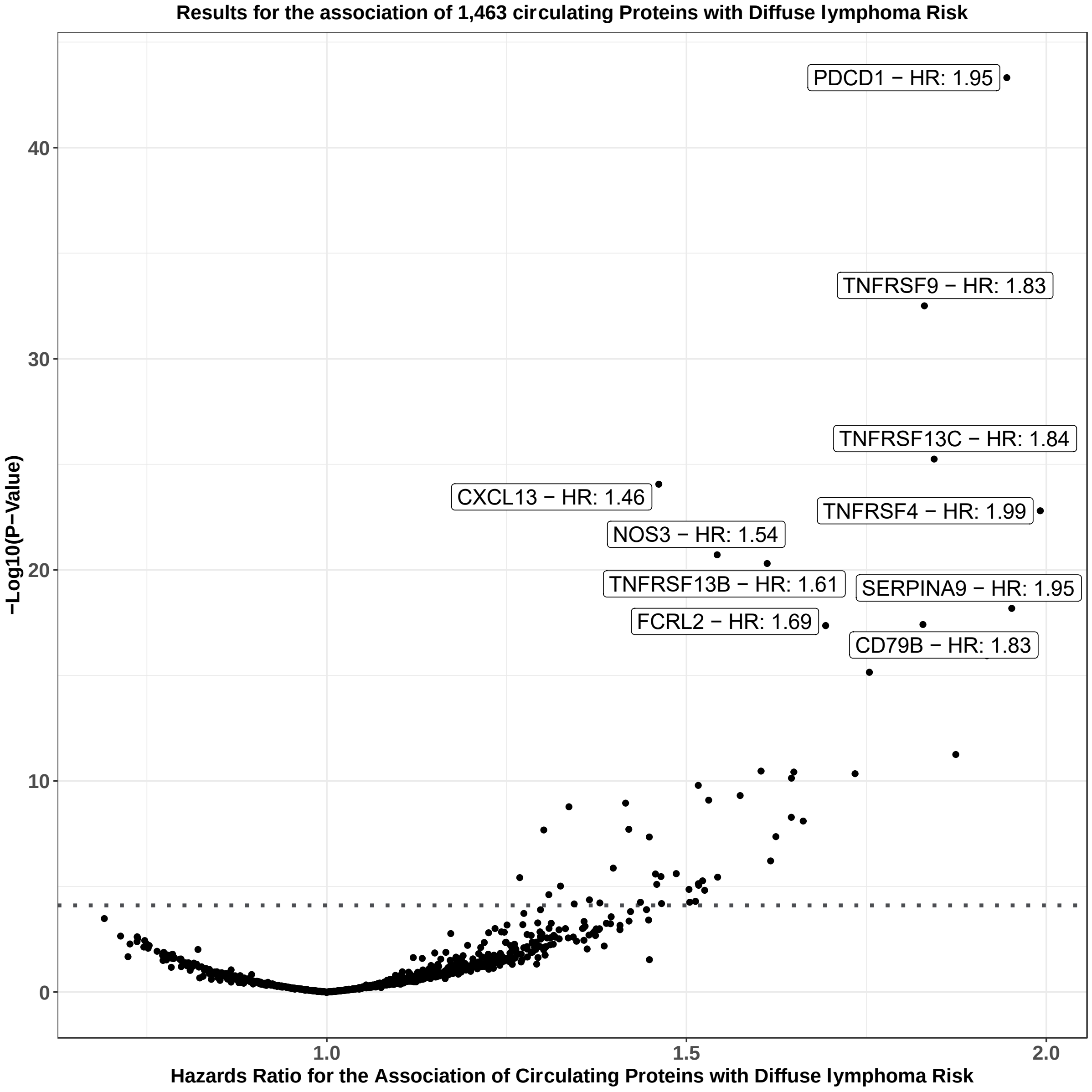

Supplementary Figure 26. Volcano plot for the prospective association of circulating proteins with risk of diffuse lymphoma

Volcano plots displaying the results from the prospective observational analyses of 1,463 proteins with risk of diffuse lymphoma. Hazard ratios per SD for cancer risk is plotted on the x-axis while –log10 p-values are plotted on the y-axis. Protein names and hazard ratios are labelled to highlight a selection of associations significant after correction for multiple testing ($p < 0.05/639$). Source data are provided as a Source Data file.

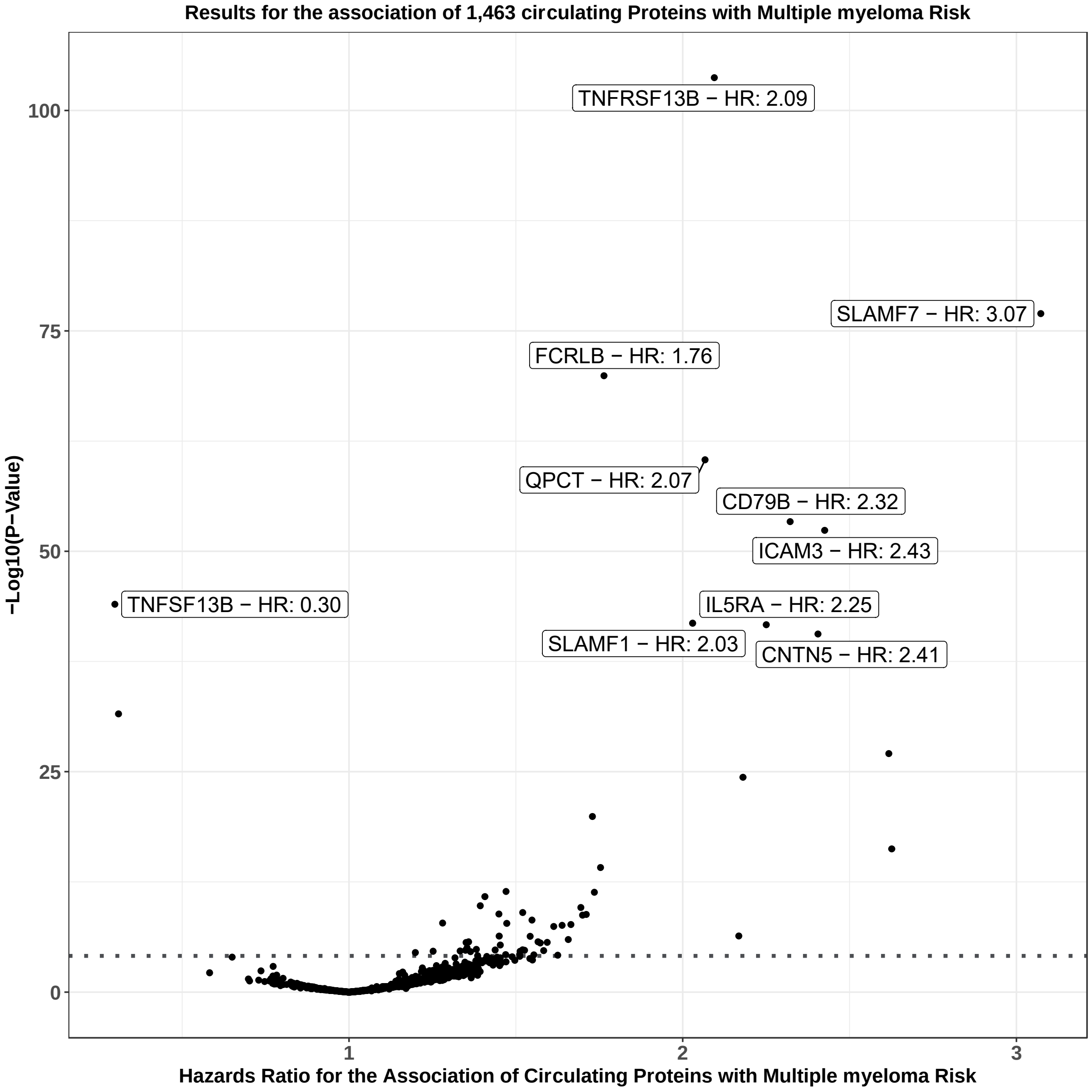

Supplementary Figure 27. Volcano plot for the prospective association of circulating proteins with risk of multiple myeloma

Volcano plots displaying the results from the prospective observational analyses of 1,463 proteins with risk of multiple myeloma. Hazard ratios per SD for cancer risk is plotted on the x-axis while –log10 p-values are plotted on the y-axis. Protein names and hazard ratios are labelled to highlight a selection of associations significant after correction for multiple testing ($p < 0.05/639$). Source data are provided as a Source Data file.

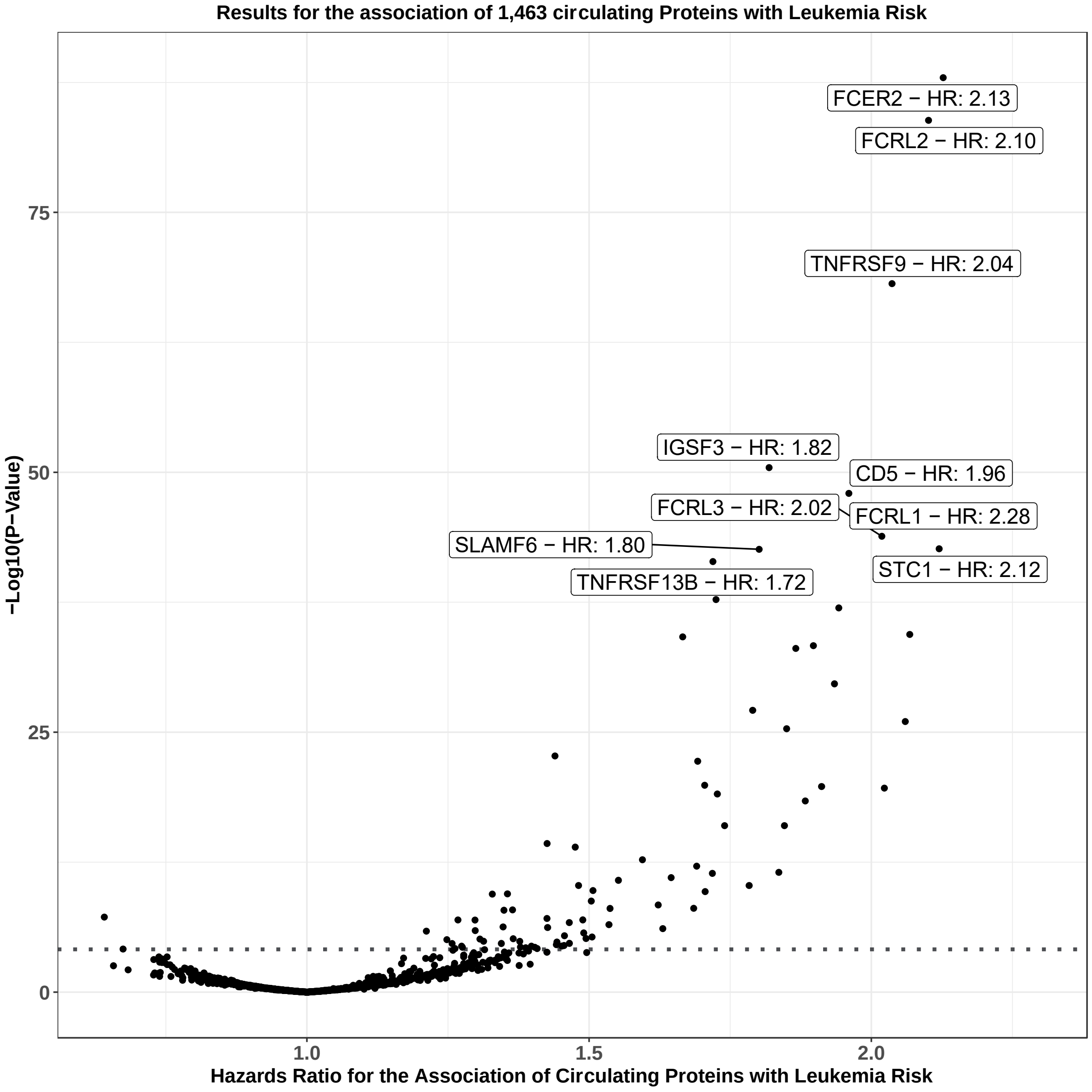

Supplementary Figure 28. Volcano plot for the prospective association of circulating proteins with risk of leukemia

Volcano plots displaying the results from the prospective observational analyses of 1,463 proteins with risk of leukemia. Hazard ratios per SD for cancer risk is plotted on the x-axis while –log10 p-values are plotted on the y-axis. Protein names and hazard ratios are labelled to highlight a selection of associations significant after correction for multiple testing ($p < 0.05/639$). Source data are provided as a Source Data file.

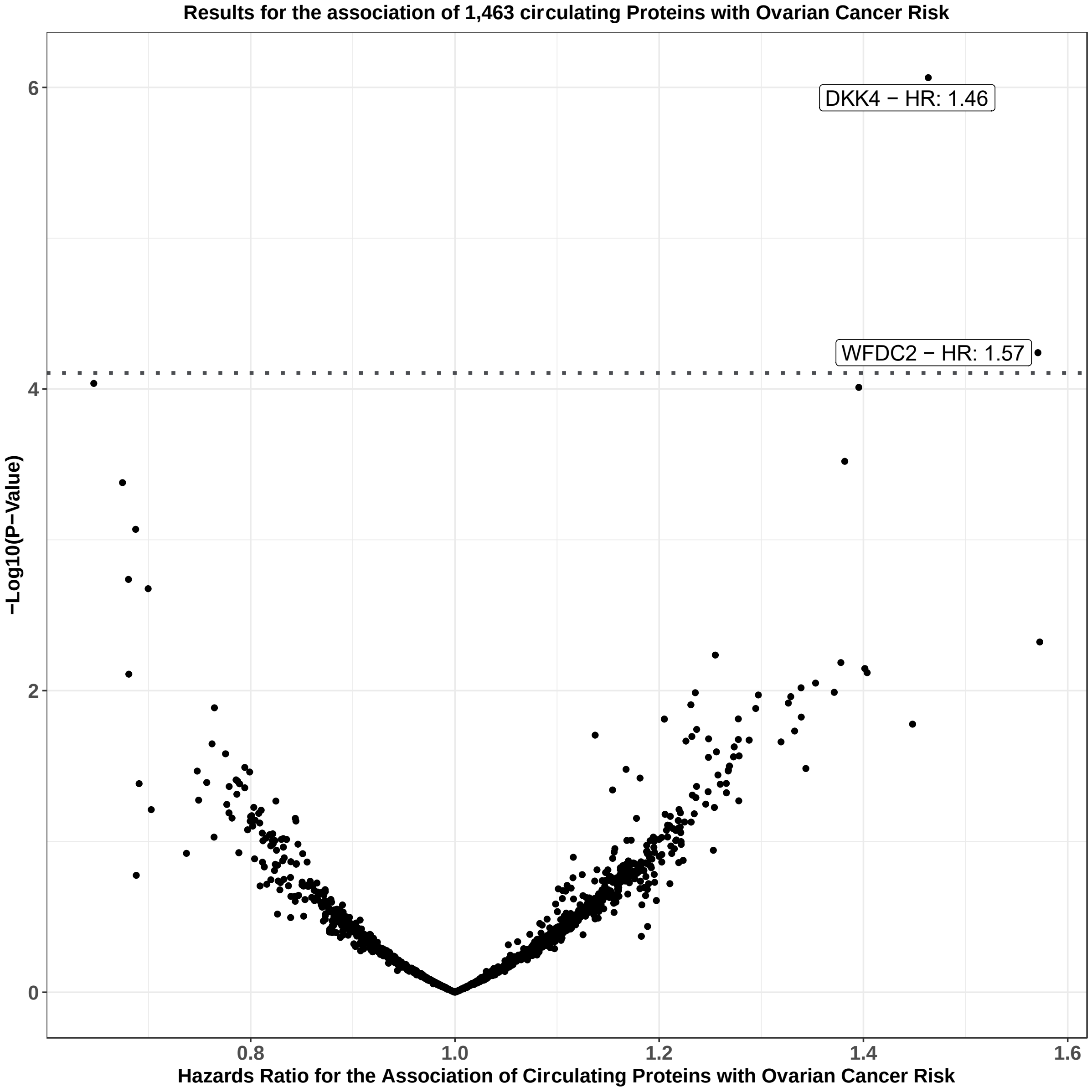

Supplementary Figure 29. Volcano plot for the prospective association of circulating proteins with risk of ovarian cancer

Volcano plots displaying the results from the prospective observational analyses of 1,463 proteins with risk of ovarian cancer. Hazard ratios per SD for cancer risk is plotted on the x-axis while –log10 p-values are plotted on the y-axis. Protein names and hazard ratios are labelled to highlight a selection of associations significant after correction for multiple testing (p < 0.05/639). Source data are provided as a Source Data file.

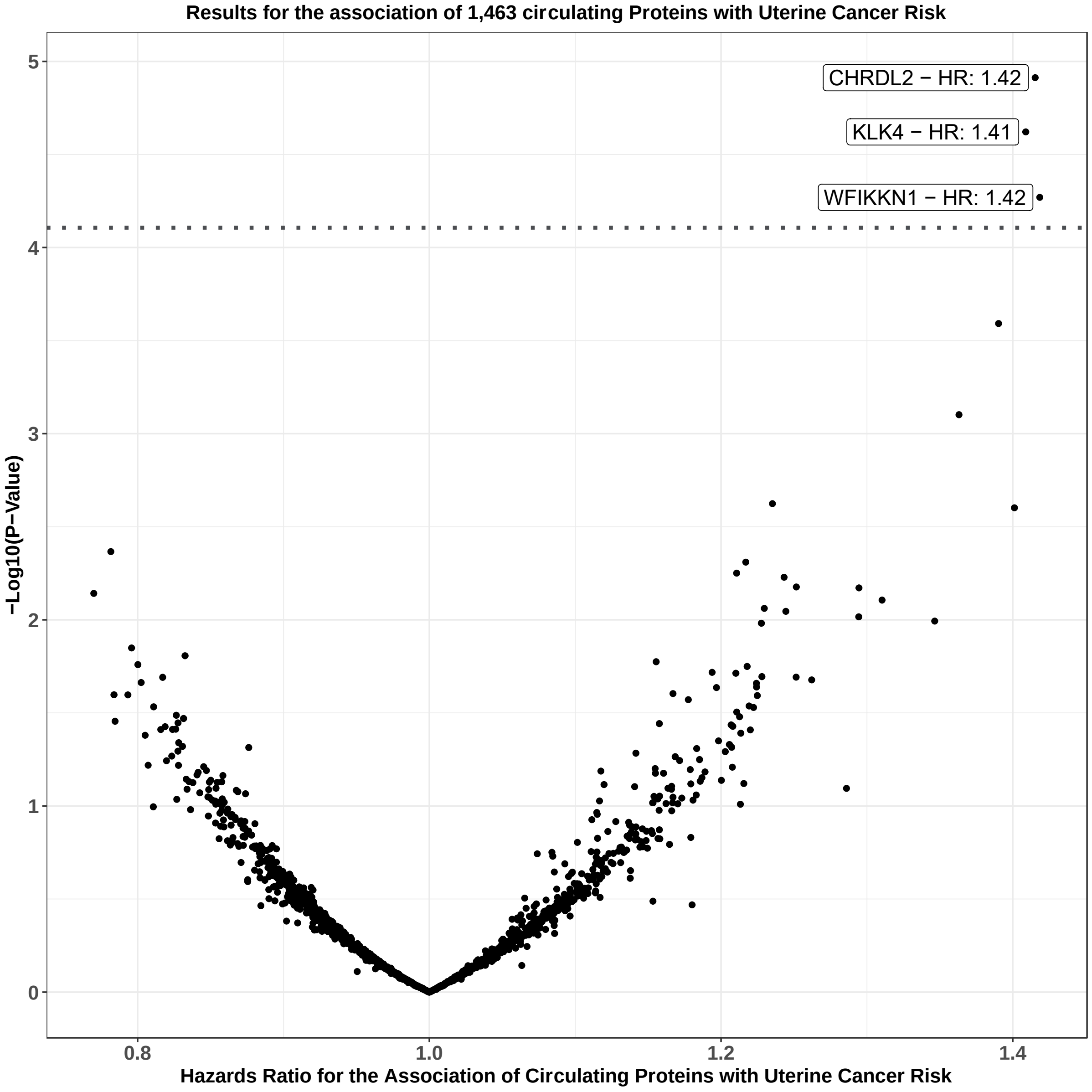

Supplementary Figure 30. Volcano plot for the prospective association of circulating proteins with risk of endometrial cancer

Volcano plots displaying the results from the prospective observational analyses of 1,463 proteins with risk of endometrial cancer. Hazard ratios per SD for cancer risk is plotted on the x-axis while –log10 p-values are plotted on the y-axis. Protein names and hazard ratios are labelled to highlight a selection of associations significant after correction for multiple testing ($p < 0.05/639$). Source data are provided as a Source Data file.

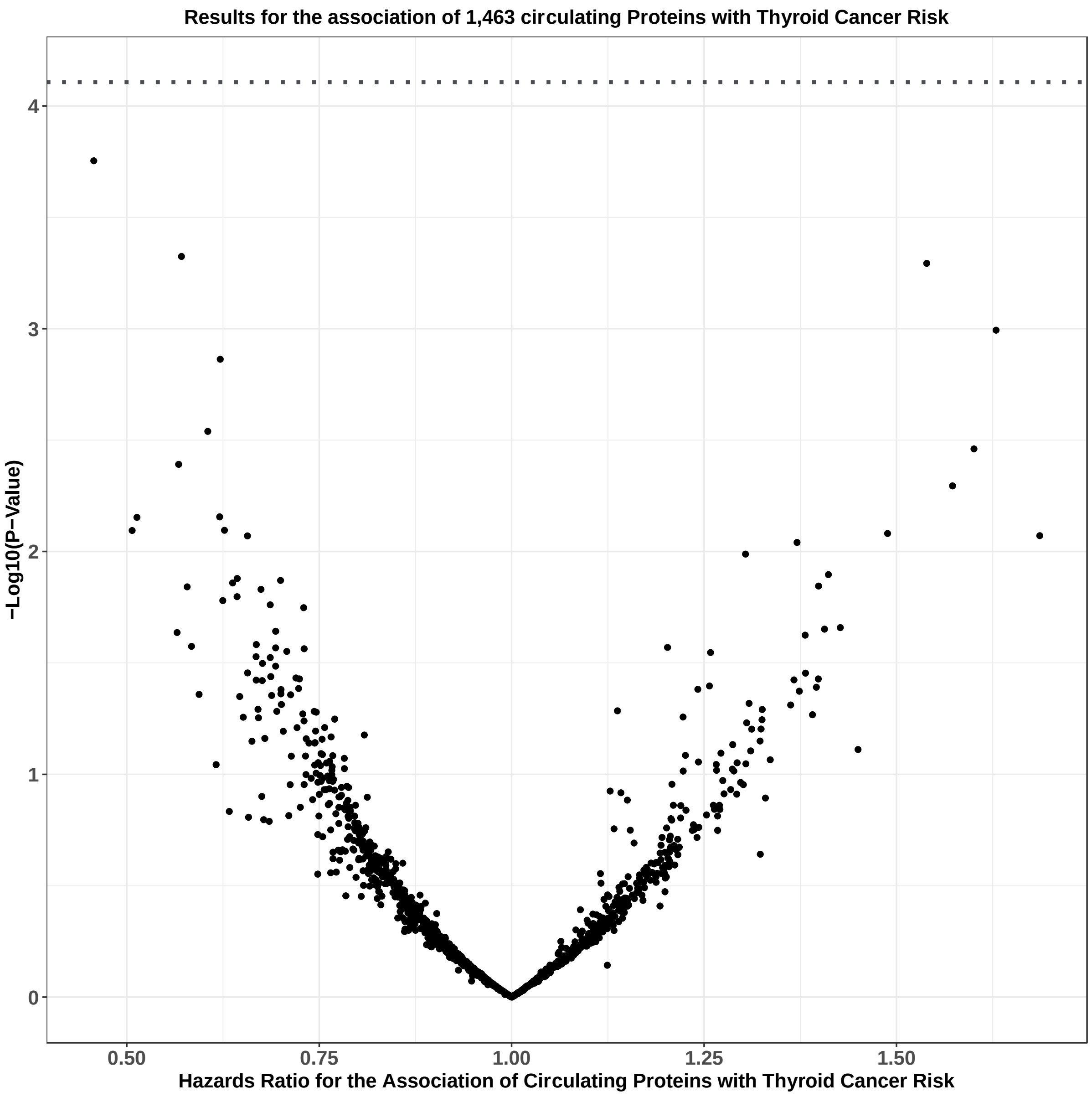

Supplementary Figure 31. Volcano plot for the prospective association of circulating proteins with risk of thyroid cancer

Volcano plots displaying the results from the prospective observational analyses of 1,463 proteins with risk of thyroid cancer. Hazard ratios per SD for cancer risk is plotted on the x-axis while –log10 p-values are plotted on the y-axis. Protein names and hazard ratios are labelled to highlight a selection of associations significant after correction for multiple testing ($p < 0.05/639$). Source data are provided as a Source Data file.
