# Supplementary material for: Identifying proteomic risk factors for cancer using prospective and exome analyses of 1463 circulating proteins and risk of 19 cancers in the UK Biobank
Source: Nat Commun. 2024 May 15;15:4010. doi: 10.1038/s41467-024-48017-6 (PMC11096312; doi:10.1038/s41467-024-48017-6)
Supplement: Supplementary file 3 — Description of Additional Supplementary Files [file 41467_2024_48017_MOESM3_ESM.docx]

**Description of Additional Supplementary Files**

File Name: Supplementary Data 1

Description: Number of cases and age of diagnosis by cancer sub-site in all participants, and in men and women

File Name: Supplementary Data 2

Description: Baseline characteristics of the UK Biobank analysis cohort, overall, by sex, and in those who developed any malignant cancer

File Name: Supplementary Data 3

Description: All protein-cancer associations from minimally adjusted, multivariable-adjusted and by time-to-diagnosis analyses (separately estimated using two-sided Cox proportional hazards regression models).

File Name: Supplementary Data 4

Description: Protein-cancer associations that pass multiple testing correction stratified by sex (separately estimated using two-sided Cox proportional hazards regression models).

File Name: Supplementary Data 5

Description: Protein-cancer associations that pass multiple test correction mapped to currently available drug

File Name: Supplementary Data 6

Description: Cis and trans single variant affecting protein-cancer associated proteins on cancer risk and exome score weights (estimated using two-sided logistic regression models).

File Name: Supplementary Data 7

Description: Association of exome score predicted protein concentration with cancer risk for protein-cancer associations that passed multiple testing correction in prospective analyses (estimated using two-sided logistic regression models).

File Name: Supplementary Data 8

Description: Protein-cancer associations that passed multiple testing correction multivariable adjusted and further adjusted for hours since last eating (estimated using two-sided Cox proportional hazards regression models).

File Name: Supplementary Data 9

Description: Summary of protein-cancer associations* that have support from one or more of long lagtime, cis-pQTL, or exome protein score analyses.

File Name: Supplementary Data 10

Description: UK Biobank exome sequencing cohort numbers per a cancer site.

Supplementary methods and figures

File Name: Supplementary Methods

Description: Pathway analyses

File Name: Supplementary Figure 1.

Description: Study design flow chart and results summary

File Name: Supplementary Figure 2.

Description: Percentage change in the log hazard ratios between fully and minimally adjusted models

File Name: Supplementary Figure 3.

Description: Pathway analysis for ENT significant protein-cancer associations across Gene-Ontology Biological Processes

File Name: Supplementary Figure 4.

Description: Pathway analysis for ENT significant protein-cancer associations across Gene-Ontology Cellular Component

File Name: Supplementary Figure 5.

Description: Pathway analysis for ENT significant protein-cancer associations across Gene-Ontology Molecular Function

File Name: Supplementary Figure 6.

Description: Descriptive summary of expression for protein identified to associate with cancer risk by cell and tissue type

File Name: Supplementary Figure 7.

Description: Volcano plot for the prospective association of circulating proteins with risk of head and neck cancer

File Name: Supplementary Figure 8.

Description: Volcano plot for the prospective association of circulating proteins with risk of oral cancer

File Name: Supplementary Figure 9.

Description: Volcano plot for the prospective association of circulating proteins with risk of oesophagus cancer

File Name: Supplementary Figure 10.

Description: Volcano plot for the prospective association of circulating proteins with risk of oesophageal adenocarcinoma

File Name: Supplementary Figure 11.

Description: Volcano plot for the prospective association of circulating proteins with risk of stomach cancer

File Name: Supplementary Figure 12.

Description: Volcano plot for the prospective association of circulating proteins with risk of colorectum cancer

File Name: Supplementary Figure 13.

Description: Volcano plot for the prospective association of circulating proteins with risk of colon cancer

File Name: Supplementary Figure 14.

Description: Volcano plot for the prospective association of circulating proteins with risk of rectal cancer

File Name: Supplementary Figure 15.

Description: Volcano plot for the prospective association of circulating proteins with risk of liver cancer

File Name: Supplementary Figure 16.

Description: Volcano plot for the prospective association of circulating proteins with risk of lung cancer

File Name: Supplementary Figure 17.

Description: Volcano plot for the prospective association of circulating proteins with risk of lung adenocarcinoma

File Name: Supplementary Figure 18.

Description: Volcano plot for the prospective association of circulating proteins with risk of lung squamous cell carcinoma

File Name: Supplementary Figure 19.

Description: Volcano plot for the prospective association of circulating proteins with risk of lung small cell carcinoma

Supplementary Figure 20.

Description: Volcano plot for the prospective association of circulating proteins with risk of breast cancer

File Name: Supplementary Figure 21.

Description: Volcano plot for the prospective association of circulating proteins with risk of prostate cancer

File Name: Supplementary Figure 22.

Description: Volcano plot for the prospective association of circulating proteins with risk of kidney cancer

File Name: Supplementary Figure 23.

Description: Volcano plot for the prospective association of circulating proteins with risk of bladder cancer

File Name: Supplementary Figure 24.

Description: Volcano plot for the prospective association of circulating proteins with risk of brain cancer

File Name: Supplementary Figure 25.

Description: Volcano plot for the prospective association of circulating proteins with risk of non-Hodgkin lymphoma

File Name: Supplementary Figure 26.

Description: Volcano plot for the prospective association of circulating proteins with risk of diffuse lymphoma

File Name: Supplementary Figure 27.

Description: Volcano plot for the prospective association of circulating proteins with risk of multiple myeloma

File Name: Supplementary Figure 28.

Description: Volcano plot for the prospective association of circulating proteins with risk of leukemia

File Name: Supplementary Figure 29.

Description: Volcano plot for the prospective association of circulating proteins with risk of ovarian cancer

File Name: Supplementary Figure 30.

Description: Volcano plot for the prospective association of circulating proteins with risk of endometrial cancer

File Name: Supplementary Figure 31.

Description: Volcano plot for the prospective association of circulating proteins with risk of thyroid cancer
