# Supplementary material for: Identifying proteomic risk factors for cancer using prospective and exome analyses of 1463 circulating proteins and risk of 19 cancers in the UK Biobank
Source: Nat Commun. 2024 May 15;15:4010. doi: 10.1038/s41467-024-48017-6 (PMC11096312; doi:10.1038/s41467-024-48017-6)
Supplement: Supplementary file 5 — Reporting Summary [file 41467_2024_48017_MOESM5_ESM.pdf]

nature portfolio

Corresponding author(s): Keren Papier

Last updated by author(s): Feb 13, 2024

Reporting Summary

Nature Portfolio wishes to improve the reproducibility of the work that we publish. This form provides structure for consistency and transparency in reporting. For further information on Nature Portfolio policies, see our Editorial Policies and the Editorial Policy Checklist.

Statistics

For all statistical analyses, confirm that the following items are present in the figure legend, table legend, main text, or Methods section.

n/a	Confirmed	
☐	☒	The exact sample size (*n*) for each experimental group/condition, given as a discrete number and unit of measurement
☐	☒	A statement on whether measurements were taken from distinct samples or whether the same sample was measured repeatedly
☐	☒	The statistical test(s) used AND whether they are one- or two-sided<br>*Only common tests should be described solely by name; describe more complex techniques in the Methods section.*
☐	☒	A description of all covariates tested
☐	☒	A description of any assumptions or corrections, such as tests of normality and adjustment for multiple comparisons
☐	☒	A full description of the statistical parameters including central tendency (e.g. means) or other basic estimates (e.g. regression coefficient) AND variation (e.g. standard deviation) or associated estimates of uncertainty (e.g. confidence intervals)
☐	☒	For null hypothesis testing, the test statistic (e.g. *F*, *t*, *r*) with confidence intervals, effect sizes, degrees of freedom and *P* value noted<br>*Give P values as exact values whenever suitable.*
☒	☐	For Bayesian analysis, information on the choice of priors and Markov chain Monte Carlo settings
☒	☐	For hierarchical and complex designs, identification of the appropriate level for tests and full reporting of outcomes
☒	☐	Estimates of effect sizes (e.g. Cohen's *d*, Pearson's *r*), indicating how they were calculated

Our web collection on statistics for biologists contains articles on many of the points above.

Software and code

Policy information about availability of computer code

Data collection	UK Biobank used the Olink Explore 1536 platform to collect all proteins data.
Data analysis	Stata version 18.1 ,R version 4.1.2, and Python were used to conduct all statistical analyses.

For manuscripts utilizing custom algorithms or software that are central to the research but not yet described in published literature, software must be made available to editors and reviewers. We strongly encourage code deposition in a community repository (e.g. GitHub). See the Nature Portfolio guidelines for submitting code & software for further information.

Data

Policy information about availability of data

All manuscripts must include a data availability statement. This statement should provide the following information, where applicable:

- Accession codes, unique identifiers, or web links for publicly available datasets
- A description of any restrictions on data availability
- For clinical datasets or third party data, please ensure that the statement adheres to our policy

Provide your data availability statement here.

Research involving human participants, their data, or biological material

Policy information about studies with human participants or human data. See also policy information about sex, gender (identity/presentation), and sexual orientation and race, ethnicity and racism.

Reporting on sex and gender	The work reported on self-reported sex
Reporting on race, ethnicity, or other socially relevant groupings	In the UK Biobank exmoe-sequence analysis, only genetic predicted Europeans were considered.
Population characteristics	This study is based on data from the UK Biobank participants, a prospective cohort of 503,317 adults aged between 39 and 71
Recruitment	UK Biobank participants were recruited between 2006 and 2010 from across the UK.
Ethics oversight	The UK Biobank study was approved by the National Information Governance Board for Health and Social Care and the National Health Service Northwest Multicenter Research Ethics Committee (06/MRE08/65).

Note that full information on the approval of the study protocol must also be provided in the manuscript.

Field-specific reporting

Please select the one below that is the best fit for your research. If you are not sure, read the appropriate sections before making your selection.

☒ Life sciences ☐ Behavioural & social sciences ☐ Ecological, evolutionary & environmental sciences

For a reference copy of the document with all sections, see nature.com/documents/nr-reporting-summary-flat.pdf

Life sciences study design

All studies must disclose on these points even when the disclosure is negative.

Sample size	The maximal analysis cohort included 44,645 participants, the genetic analysis contained the full 450,000 UKBB exome-seq cohort
Data exclusions	Of the 54,306 participants who were selected for proteomic profiling as part of the UK Biobank Pharma Proteomics Project , 1,601 had samples that did not pass quality control. From the remaining 52,705, We further excluded 2,969 participants due to cancer diagnosis at or prior to baseline (except non–melanoma skin cancer C44), 37 whose self–reported sex did not match their genetic sex, 242 who had missing information on height or weight, 2,113 who were currently using hormone replacement therapy or oral contraceptives, and 2,709 who reported having diabetes at baseline. Individuals that withdrawnfor the UKBB were removed from the UKBB exome cohort. Genetic predicted Non-European individuals were removed from the UKBB exome.
Replication	Replication will be required on an external dataset. Some cancers are rare and we therefore lack power to conduct an internal validation by splitting the dataset.
Randomization	Not relevant because this is an observational cohort study
Blinding	Not relevant because this is an observational cohort study

Reporting for specific materials, systems and methods

We require information from authors about some types of materials, experimental systems and methods used in many studies. Here, indicate whether each material, system or method listed is relevant to your study. If you are not sure if a list item applies to your research, read the appropriate section before selecting a response.

Materials & experimental systems

n/a	Involved in the study	
☒	☐	Antibodies
☒	☐	Eukaryotic cell lines
☒	☐	Palaeontology and archaeology
☒	☐	Animals and other organisms
☐	☒	Clinical data
☒	☐	Dual use research of concern
☒	☐	Plants

Methods

n/a	Involved in the study	
☒	☐	ChIP-seq
☒	☐	Flow cytometry
☒	☐	MRI-based neuroimaging

Plants

Seed stocks	NA
Novel plant genotypes	NA
Authentication	NA
